# Associations of the *PON1* rs662 polymorphism with circulating oxidized low-density lipoprotein and lipid levels: a systematic review and meta-analysis

**DOI:** 10.1186/s12944-018-0937-8

**Published:** 2018-12-13

**Authors:** Zhi Luo, Lijun Pu, Irfan Muhammad, Yun Chen, Xiaoqian Sun

**Affiliations:** Department of Cardiology, Affiliated Hospital of North Sichuan Medical College, Nanchong, 637000 People’s Republic of China

**Keywords:** Paraoxonase 1, Oxidized low-density lipoprotein, Lipid, Polymorphism, Coronary heart disease

## Abstract

**Background:**

Several meta-analyses have demonstrated that the rs662 polymorphism in Paraoxonase 1 gene (*PON1*) gene is associated with coronary heart disease (CHD). However, it is still uncertain whether this polymorphism is associated with the plasma levels of oxidized low-density lipoprotein (Ox-LDL) and lipids. This meta-analysis is aimed to clarify the relationships between the rs662 polymorphism and plasma levels of Ox-LDL and lipids.

**Methods:**

By searching in PubMed, Google Scholar, Web of Science, Cochrane Library, Wanfang, VIP and CNKI databases, 5 studies (1369 subjects) and 85 studies (46,740 subjects) were respectively identified for Ox-LDL association analysis and lipid association analysis. Standardized mean difference (SMD) was used to estimate the effects of the rs662 polymorphism on plasma Ox-LDL and lipid levels.

**Results:**

The carriers of the variant R allele had higher levels of Ox-LDL (SMD = 0.23, 95% CI = 0.10–0.36, *P* <  0.01), triglyceride (TG) (SMD = 0.06, 95% CI = 0.01–0.11, *P* = 0.02), total cholesterol (TC) (SMD = 0.04, 95% CI = 0.00–0.07, *P* = 0.05) and low-density lipoprotein cholesterol (LDL-C) (SMD = 0.04, 95% CI = 0.00–0.08, *P* = 0.04) than the non-carriers.

**Conclusions:**

This meta-analysis suggests that the association between the *PON1* rs662 polymorphism and CHD may partly be mediated by abnormal Ox-LDL and lipid levels caused by the R allele.

**Electronic supplementary material:**

The online version of this article (10.1186/s12944-018-0937-8) contains supplementary material, which is available to authorized users.

## Introduction

Coronary heart disease (CHD) is currently the leading cause of death in developed and some developing countries like China [1]. CHD is a multifactorial disease with a number of risk factors being identified in the past few decades. Among these risk factors, the increase in circulating oxidized low-density lipoprotein (Ox-LDL) and dyslipidemia were widely reported with regard to their important roles in the occurrence and development of CHD [2, 3]. Ox-LDL, formed by oxidative modification of low density lipoprotein, is thought to play a key role in atherogenesis and induce a wide range of biological effects on smooth muscle cells. Dyslipidemia is a state of abnormal amounts of lipids (e.g., triglycerides, cholesterol and/or phospholipids) in the blood, and is characterized by increased levels of triglycerides (TG), total cholesterol (TC) and low-density lipoprotein cholesterol (LDL-C), and/or decreased level of high-density lipoprotein cholesterol (HDL-C) in circulation. Intensive efforts have been made in the scientific community to investigate the associations of the genetic polymorphisms in specific genes with the risk factors for CHD, but the results were inconsistent and inconclusive. It is difficult to successfully identify the genetic polymorphisms being associated with the risk factors for CHD due to various reasons such as small sample sizes and ethnic differences.

In humans, PON has three isoforms: paraoxonase 1 (PON1), paraoxonase 2 (PON2) and paraoxonase 3 (PON3). Among them, PON1 is a calcium-dependent multifunctional enzyme that connects metabolisms of homocysteine (Hcy) and lipoproteins. First of all, PON1 plays a key role in Hcy metabolism by irreversibly catalyzing the conversion of homocysteine thiolactone (Hcy T) to Hcy. By detoxifying Hcy T, the PON1 would protect proteins against homocysteinylation, a potential risk factor for CHD [4]. Secondly, PON1 is a HDL-associated enzyme that inhibits the oxidation of LDL [5] and HDL [6] as well as promote cholesterol efflux from macrophage foam cells [7]. PON1 has been reported to be associated with abnormal lipid levels and CHD risk [8, 9]. Shih et al. [10] reported that PON1 (−/−) mice had significantly higher levels of plasma Ox-LDL and Ox-HDL compared with PON1 (+/+) mice. When fed on a high-fat diet, PON1 (−/−) mice were more susceptible to atherosclerosis than PON1 (+/+) mice.

The *PON1* gene is located on the long arm of human chromosome 7 (7q21.3–22.1), and it contains 9 exons and 8 introns. There are several polymorphisms in the *PON1* gene. The rs662 polymorphism (also known as Q192R) is located in exon 6 of the *PON1* gene and formed by a transition from adenine (A) to guanine (G). Accordingly, the 192th genetic code is changed from CAA to CGA, resulting in the replacement of glutamine (Gln) by arginine (Arg) in the *PON1* polypeptide. A large number of researches have investigated the associations of the rs662 polymorphism with plasma Ox-LDL and lipid levels. In some of these studies, the R allele of the rs662 polymorphism was reported to be associated with increased levels of Ox-LDL [11, 12], TG [9, 13, 14], TC [8, 9, 15] and LDL-C [8, 9, 15–17], and reduced levels of HDL-C [18–20]. However, the results obtained from other studies did not support these findings [21–23]. Hence, a meta-analysis is required to clarify the relationships of the rs662 polymorphism with Ox-LDL and lipids.

In this study, a systematic review and meta-analysis was performed based on previous publications to investigate the associations of the rs662 polymorphism with Ox-LDL and lipid levels. Our analysis results can provide an opportunity to unveil the interrelationships among the rs662 polymorphism, Ox-LDL, dyslipidemia and susceptibility to CHD.

## Methods

### Literature search

The articles published until September 2018 on the associations of the rs662 polymorphism with circulating Ox-LDL and lipid levels were identified. The languages of the articles were limited to English and Chinese. A comprehensive search was conducted and nine electronic databases were searched to identify all relevant articles. The databases were as follows: PubMed, Embase, Baidu Scholar, Google Scholar, Web of Science, Cochrane Library, Wanfang, CBM and CNKI. The following keywords were used: (“Paraoxonase 1” or “PON1”), (“Q192R” or “rs662” or “Gln192Arg”), (“polymorphism” or “mutation” or “variant”), (“Ox-LDL” or “oxidized low-density lipoprotein”) and (“plasma lipid” or “blood lipid” or “serum lipid”).

### Inclusion and exclusion criteria

The inclusion criteria for the meta-analysis are as follows: 1) studies which presented the genotype and allele frequencies of the rs662 polymorphism; 2) studies in which mean Ox-LDL and mean lipids with standard deviation (SD) or standard error (SE) according to the rs662 genotypes were available; 3) studies which reported at least one of the five variables, i.e., Ox-LDL, TG, TC, LDL-C and HDL-C. All references cited by the included articles were reviewed to check the published work which was not indexed by PubMed, Embase, Baidu Scholar, Google Scholar, Web of Science, Cochrane Library, Wanfang, CBM and CNKI. Reports with incomplete data, studies based on pedigree data, case reports, review articles, abstracts and animal studies were excluded from the meta-analysis. Pre-intervention data were used for interventional studies.

### Data extraction

Data were extracted from each study by using a structured data collection form and by two investigators independently according to the pre-specified selection criteria. Decisions were compared and disagreements about study selection were resolved by consensus or involving a third investigator. For the overlapping articles, only the publications that presented the most detailed information were included. In this meta-analysis, the data extracted from each of the included studies are as follows: first author, year of publication, age, ethnicity, gender, health status, type of study, lipid assay method, sample size, and mean with SD or SE according to the rs662 genotypes. If data in a study were unconvincing, we attempted to contact the corresponding or first author by e-mail or telephone.

### Data analysis

Statistical analysis was performed using STATA version 12.0 (Stata Corporation LP, College Station, TX, USA). All the tests were two-sided and a *P*-value of less than 0.05 for any test or model was considered to be statistically significant. The pooled standardized mean difference (SMD) with 95% confidence interval (CI) was used to assess the strength of the associations between the rs662 polymorphism and plasma levels of Ox-LDL and lipids. Random-effect model (DerSimonian-Laird method) was used to evaluate the results if heterogeneity among the included studies was significant (*I*^2^ > 50%). Otherwise, Fixed-effect model (Mantel-Haenszel method) was used [24]. Heterogeneity was investigated by Cochran’s χ^2^-based Q-statistic, and Galbraith plots were used to detect the potential sources of heterogeneity. SMD values were recalculated after excluding the studies with heterogeneity. Subgroup analyses were performed according to gender, ethnicity and health status. Ethnic subgroup was defined as Caucasian, Asian, African, and the subjects of other ethnic origin. Health status was defined as CHD, T2DM and Hyperlipidemia. HWE was assessed by Fisher’s exact test. Publication bias was tested by Begg’s funnel plot and Egger’s test [25].

## Result

### Characteristics of the included studies

**Fig. 1:**
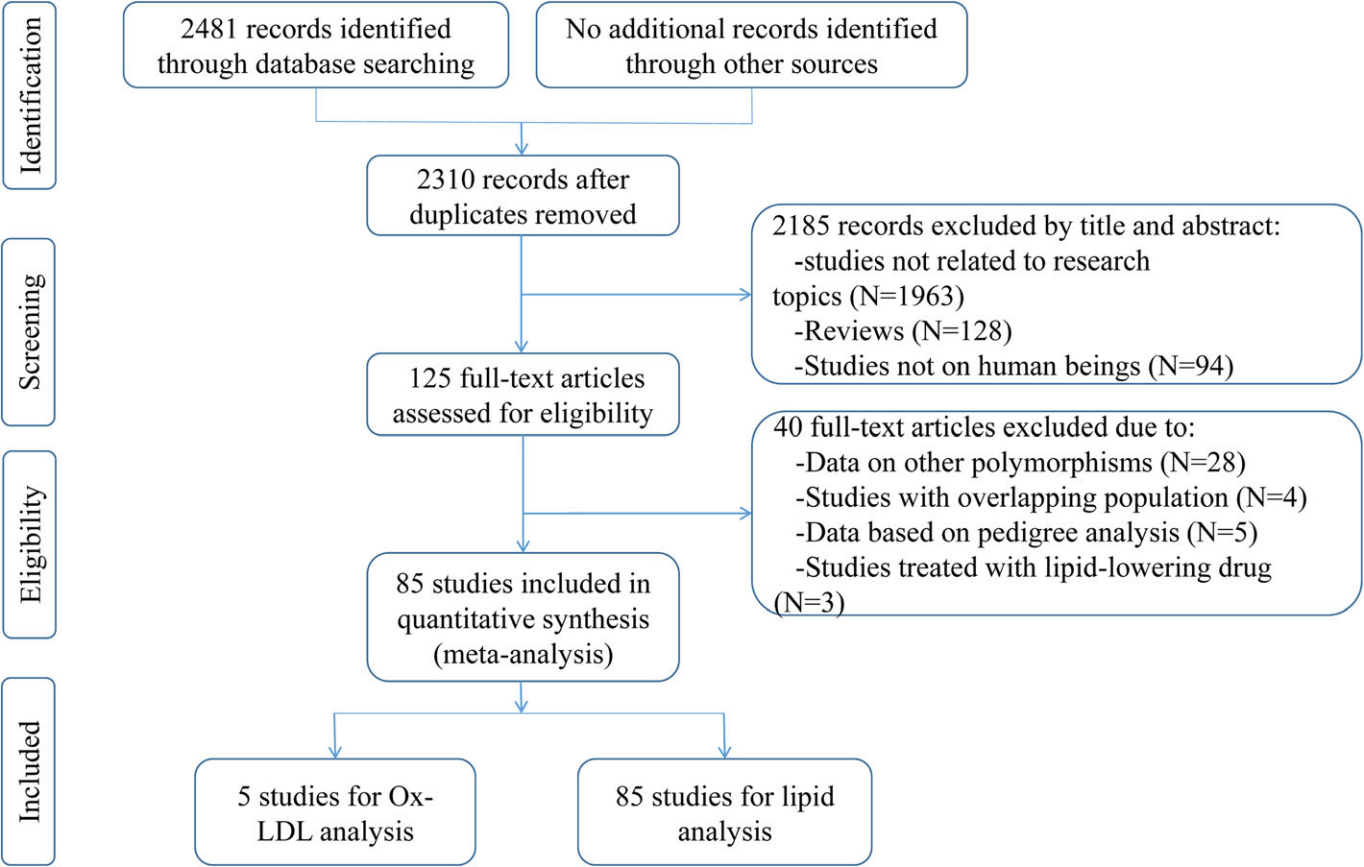
Flow diagram of the study selection process

Initial search of the databases yielded 2481 articles. Two thousand one hundred and eighty-five studies were excluded according to titles and abstracts. Then full-text articles were retrieved and assessed on the basis of the inclusion criteria. Forty studies were ineligible for the following reasons: 28 studies presented data for other polymorphisms, 4 studies had subjects overlapping with other publications, 5 studies were based on pedigree analysis, and 3 studies were treated with lipid-lowering drugs. In the end, 85 studies were selected for this meta-analysis (Fig. 1). Of them, 5 studies (1369 subjects) were included in the Ox-LDL association analysis and 85 studies (46,740 subjects) were included in the lipid association analysis. The references for the studies included in the present meta-analysis were listed in Additional file 1.

The characteristics of the studies included in the Ox-LDL and lipids association analysis were summarized in Additional file 2: Table S1. The plasma Ox-LDL levels by the genotypes of the rs662 polymorphism were presented in Additional file 2: Table S2. One study and 4 studies were involved in Caucasians and Asians, respectively. The plasma lipid levels by the genotypes of the rs662 polymorphism were presented in Additional file 2: Table S3. Forty-three studies, 25 studies, 5 studies and 12 studies were involved in Caucasians, Asians, Africans and the subjects of other ethnic origins, respectively.

### Association of the rs662 polymorphism with ox-LDL

**Fig. 2:**
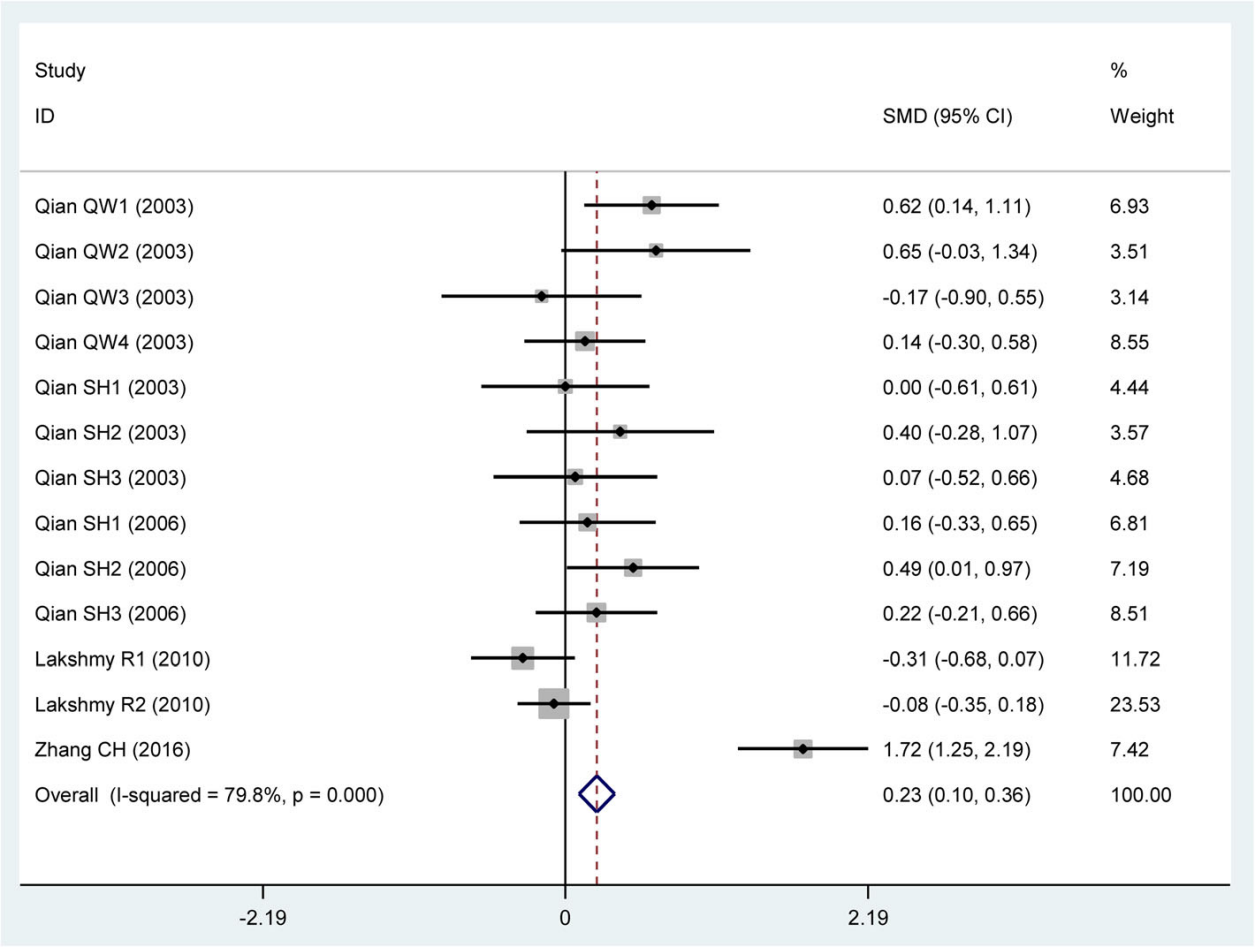
Forest plot of the meta-analysis between the *PON1* rs662 polymorphism and circulating oxidized low-density lipoprotein (Ox-LDL) levels

The carriers of the variant R allele had higher levels of Ox-LDL (SMD = 0.23, 95% CI = 0.10–0.36, *P* <  0.01) (Table 1, Fig. 2) than the non-carriers. When the analysis was limited to the studies in HWE, the association between the rs662 polymorphism and Ox-LDL was also significant (SMD = 0.32, 95% CI = 0.12–0.53, *P* <  0.01) (Table 1).

**Table 1:** Meta-analysis between the *PON1* rs662 polymorphism and Ox-LDL levels

Groups or subgroups	Comparisons (Subjects)	SMD (95% CI)	*P*_Heterogeneity_	*P*_SMD_
Ox-LDL				
All	13 (1369)	0.23 (0.10–0.36)	< 0.01	< 0.01
Studies in HWE	3 (426)	0.32 (0.12–0.53)	< 0.01	< 0.01
Caucasian	2 (345)	−0.16 (− 0.37–0.06)	0.34	0.15
Asian	11 (1024)	0.44 (0.28–0.60)	< 0.01	< 0.01
CHD	3 (274)	0.39 (0.12–0.66)	< 0.01	< 0.01
Healthy or control	4 (518)	0.03 (−0.16–0.22)	0.63	0.74

*PON1* Paraoxonase 1 gene, *SMD* standardized mean difference, *95% CI* 95% confidence interval, *HWE* Hardy-Weinberg equilibrium, *Ox-LDL* oxidized low density lipoprotein, *CHD* coronary heart disease

The subgroup analyses stratified by the characteristics of the subjects were performed, and the results showed that the rs662 polymorphism was significantly associated with Ox-LDL levels in Asians and CHD patients (Table 1).

### Associations of the rs662 polymorphism with plasma lipid levels

**Fig. 3:**
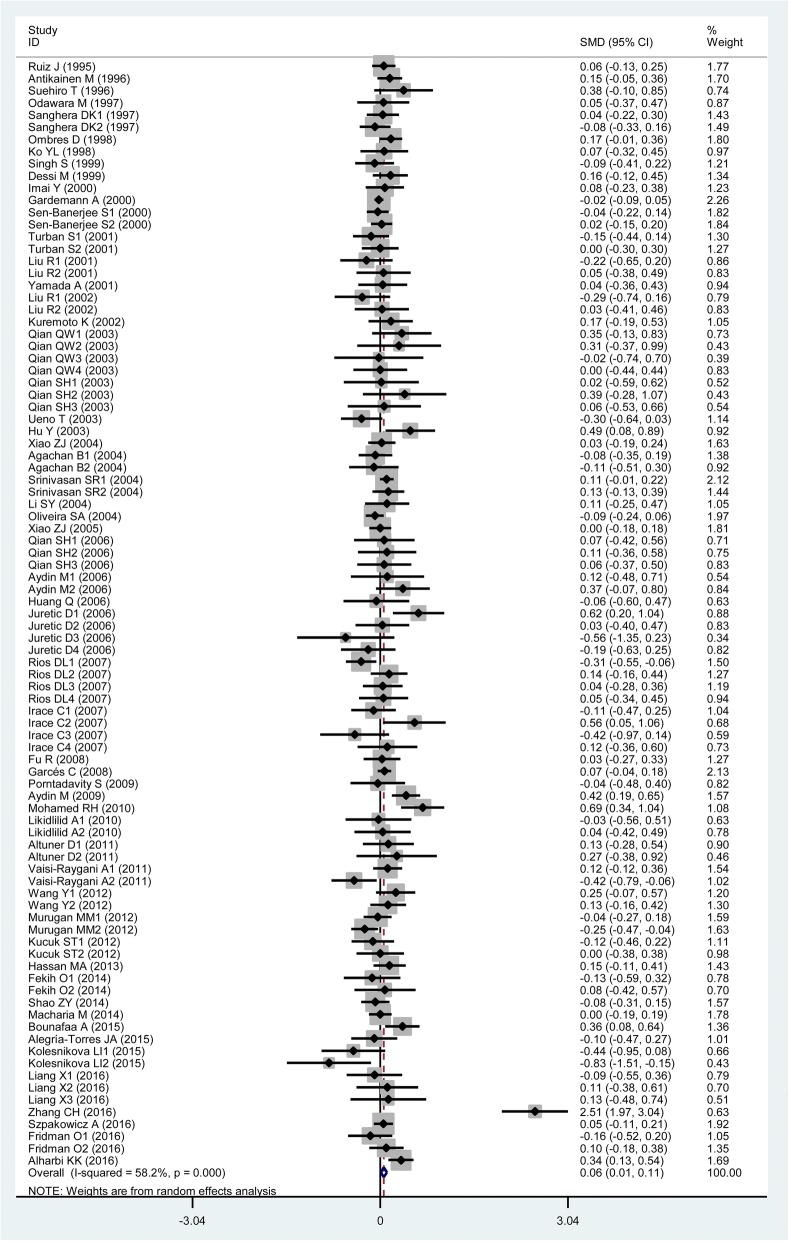
Forest plot of the meta-analysis between the *PON1* rs662 polymorphism and plasma triglycerides (TG) levels

**Fig. 4:**
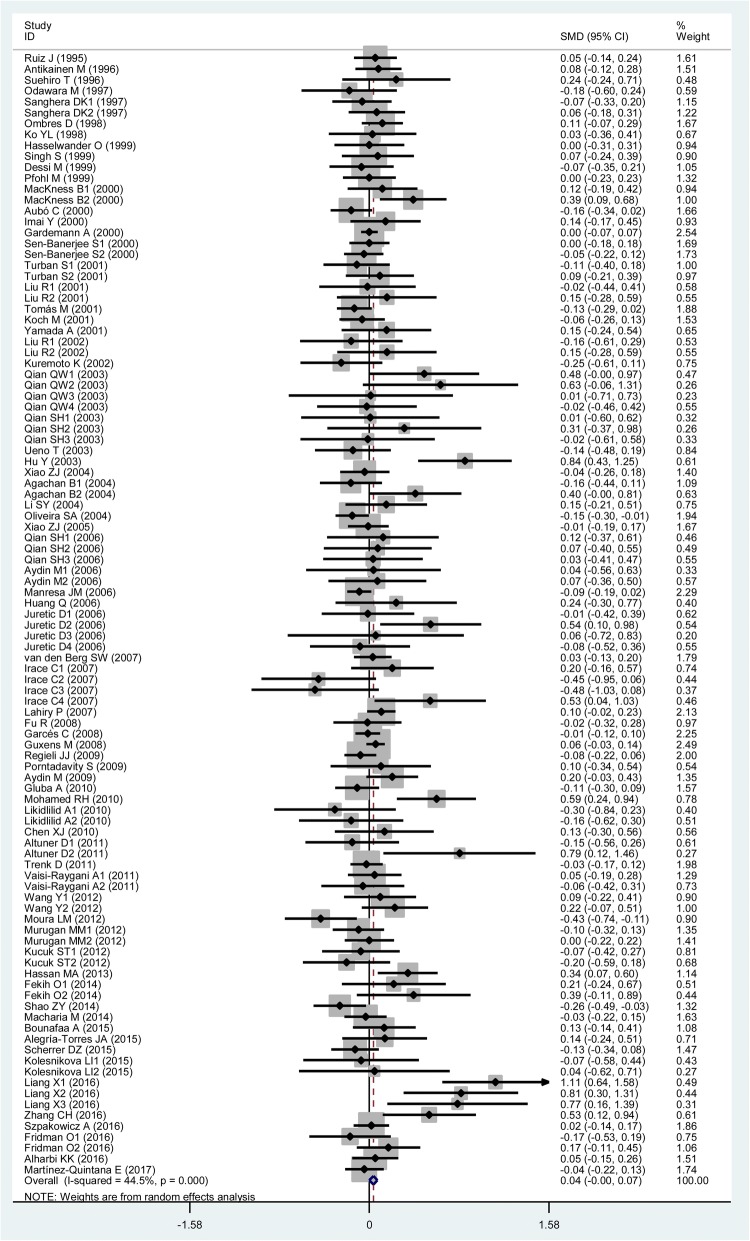
Forest plot of the meta-analysis between the *PON1* rs662 polymorphism and plasma total cholesterol (TC) levels

**Fig. 5:**
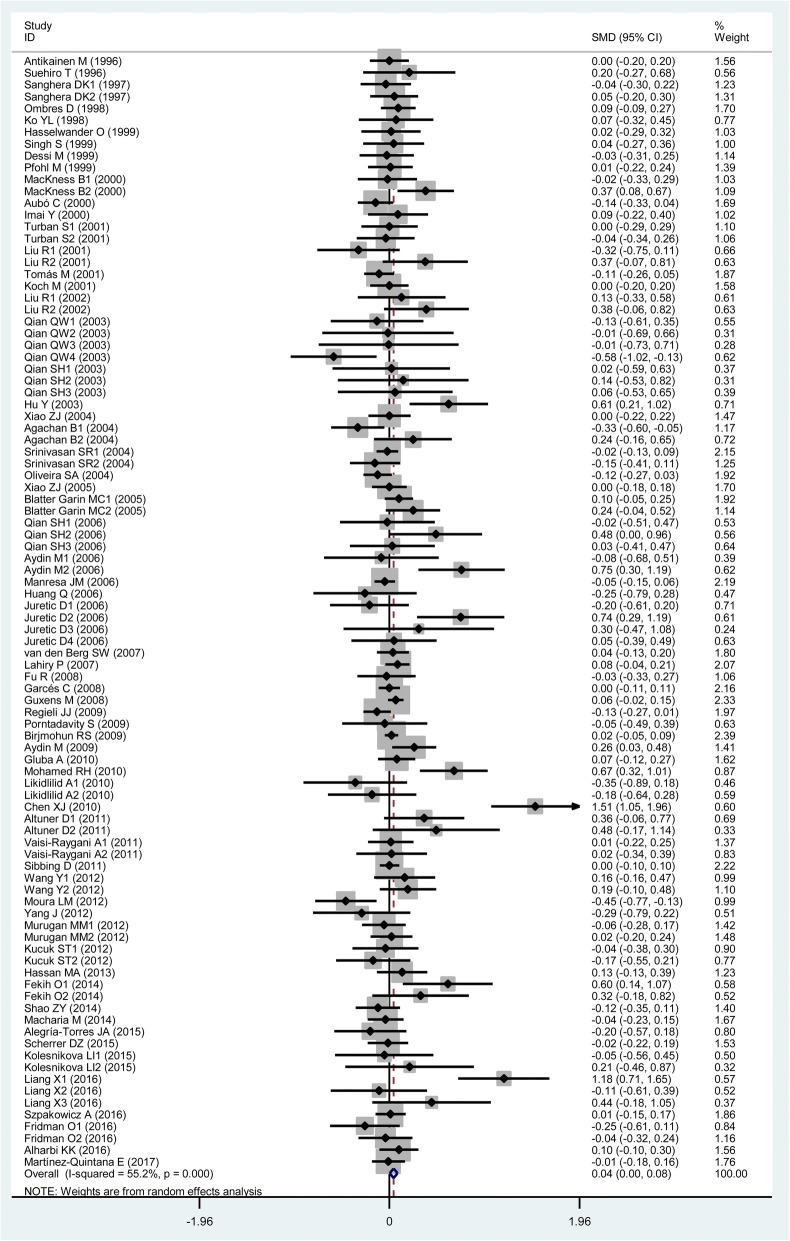
Forest plot of the meta-analysis between the *PON1* rs662 polymorphism and plasma low-density lipoprotein cholesterol (LDL-C) levels

**Fig. 6:**
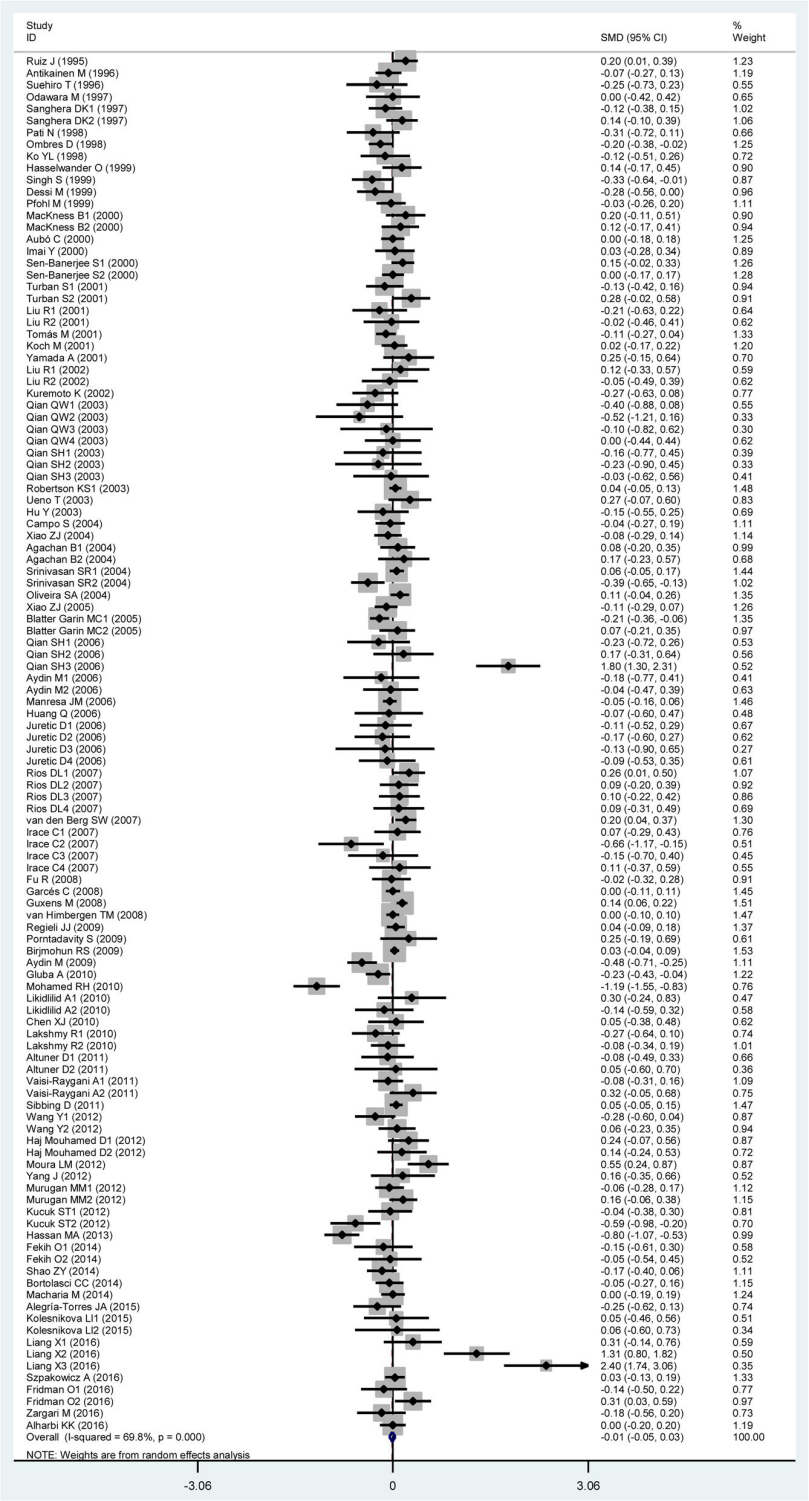
Forest plot of the meta-analysis between the *PON1* rs662 polymorphism and plasma high-density lipoprotein cholesterol (HDL-C) levels

The outcomes of the analysis on all comparisons showed that the R allele carriers had higher levels of TG (SMD = 0.06, 95% CI = 0.01–0.11, *P* = 0.02), TC (SMD = 0.04, 95% CI = 0.00–0.07, *P* = 0.05) and LDL-C (SMD = 0.04, 95% CI = 0.00–0.08, *P* = 0.04) than the non-carriers (Table 2, Figs. 3, 4 and 5), and that there was no association detected between the rs662 polymorphism and plasma levels of HDL-C (SMD = −0.01, 95% CI = −0.05–0.04, *P* = 0.68) (Table 2, Fig. 6). When the analysis was limited to the studies in HWE, the R allele carriers had higher level of TG (SMD = 0.06, 95% CI = 0.00–0.12, *P* = 0.04) and LDL-C (SMD = 0.04, 95% CI = 0.00–0.09, *P* = 0.04) than the non-carriers (Table 2). No associations were found between the rs662 polymorphism and plasma levels of TC (SMD = 0.03, 95% CI = − 0.01-0.07, *P* = 0.13) and HDL-C (SMD = − 0.01, 95% CI = − 0.05-0.04, *P* = 0.78) (Table 2).

**Table 2:** Meta-analysis between the *PON1* rs662 polymorphism and plasma lipid levels

Groups or subgroups	Comparisons (Subjects)	SMD (95% CI)	*P*_Heterogeneity_	*P*_SMD_
TG				
All	91 (24,704)	0.06 (0.01–0.11)	< 0.01	0.02
Studies in HWE	64 (20,340)	0.06 (0.00–0.12)	< 0.01	0.04
Male	5 (3322)	−0.01 (− 0.26–0.24)	< 0.01	0.93
Female	6 (1210)	0.10 (−0.04–0.25)	0.73	0.15
Caucasian	32 (11,555)	0.06 (−0.01–0.13)	< 0.01	0.08
Asian	35 (7659)	0.12 (0.00–0.23)	< 0.01	0.04
African	6 (1880)	0.03 (−0.09–0.15)	0.94	0.58
Other ethnicity	18 (3610)	−0.00 (− 0.10–0.10)	0.02	0.98
CHD	16 (3912)	0.10 (0.04–0.17)	< 0.01	< 0.01
T2DM	10 (1715)	0.18 (0.07–0.28)	0.03	< 0.01
Hyperlipidemia	3 (296)	−0.13 (− 0.43–0.17)	0.66	0.39
Healthy or control	36 (8693)	0.03 (−0.02–0.08)	0.30	0.22
TC				
All	103 (33,696)	0.04 (0.00–0.07)	< 0.01	0.05
Studies in HWE	75 (27,792)	0.03 (−0.01–0.07)	< 0.01	0.13
Male	5 (4354)	−0.03 (− 0.09–0.03)	0.58	0.26
Female	4 (914)	0.19 (−0.03–0.40)	0.24	0.09
Caucasian	46 (19,714)	0.01 (−0.03–0.05)	0.01	0.65
Asian	36 (7869)	0.14 (0.05–0.24)	< 0.01	< 0.01
African	3 (1051)	0.10 (−0.15–0.35)	0.22	0.42
Other ethnicity	18 (5062)	−0.02 (− 0.10–0.07)	0.07	0.68
CHD	19 (5995)	0.01 (−0.05–0.06)	0.58	0.84
T2DM	12 (2213)	0.12 (−0.04–0.28)	< 0.01	0.14
Hyperlipidemia	3 (296)	−0.16 (− 0.46–0.14)	0.75	0.29
Healthy or control	40 (11,168)	0.07 (0.01–0.14)	< 0.01	0.04
LDL-C				
All	94 (34,757)	0.04 (0.00–0.08)	< 0.01	0.04
Studies in HWE	73 (31,416)	0.04 (0.00–0.09)	< 0.01	0.04
Male	4 (1570)	0.10 (−0.22–0.02)	0.72	0.08
Female	4 (914)	0.26 (0.01–0.51)	0.12	0.04
Caucasian	41 (21,056)	0.01 (−0.03–0.05)	0.01	0.49
Asian	31 (7034)	0.13 (0.01–0.26)	< 0.01	0.04
African	4 (1605)	0.12 (−0.17–0.40)	0.02	0.43
Other ethnicity	18 (5062)	0.02 (−0.08–0.12)	0.01	0.69
CHD	16 (5148)	−0.01 (− 0.06–0.05)	0.45	0.85
T2DM	8 (1446)	0.36 (0.03–0.69)	< 0.01	0.03
Hyperlipidemia	4 (399)	−0.09 (− 0.34–0.16)	0.56	0.48
Healthy or control	39 (12,230)	0.06 (0.00–0.13)	< 0.01	0.05
HDL-C				
All	116 (40,336)	−0.01 (−0.05–0.04)	< 0.01	0.68
Studies in HWE	87 (34,743)	−0.01 (− 0.05–0.04)	< 0.01	0.78
Male	7 (3729)	0.03 (−0.05–0.11)	0.28	0.51
Female	7 (2724)	−0.01 (− 0.09–0.07)	0.63	0.78
Caucasian	54 (26,409)	−0.03 (− 0.08–0.02)	< 0.01	0.22
Asian	35 (7802)	0.08 (−0.06–0.22)	< 0.01	0.25
African	8 (2180)	−0.01 (− 0.17–0.14)	0.09	0.90
Other ethnicity	19 (3945)	−0.02 (− 0.12–0.09)	< 0.01	0.76
CHD	18 (5355)	−0.06 (− 0.12−− 0.01)	0.01	0.02
T2DM	13 (2328)	-0.01 (− 0.09–0.08)	0.62	0.91
Hyperlipidemia	3 (296)	0.11 (−0.19–0.41)	0.55	0.48
Healthy or control	47 (15,342)	0.02 (−0.01–0.06)	< 0.01	0.21

*PON1* Paraoxonase 1 gene, *SMD* standardized mean difference, *95% CI* 95% confidence interval, *HWE* Hardy-Weinberg equilibrium, *TG* triglyceride, *TC* total cholesterol, *LDL-C* low-density lipoprotein cholesterol, *HDL-C* high-density lipoprotein cholesterol, *CHD* coronary heart disease, *T2DM* type 2 diabetes mellitus

Then the subgroup analysis stratified by the characteristics of the subjects was performed. The significant association of the rs662 polymorphism with higher levels of LDL-C (SMD = 0.26, 95% CI = 0.01–0.51, *P* = 0.04) was detected in the females, but not in the males. The significant associations of the rs662 polymorphism with higher levels of TG (SMD = 0.12, 95% CI = 0.00–0.23, *P* = 0.04), TC (SMD = 0.14, 95% CI = 0.05–0.24, *P* <  0.01) and LDL-C (SMD = 0.13, 95% CI = 0.01–0.26, *P* = 0.04) were detected in Asians, but not in the other ethnicities. When health status was taken into account, the significant association of the rs662 polymorphism with higher levels of TG was detected in CHD patients and T2DM patients, but not in hyperlipidemia patients and healthy subjects; the significant association of the rs662 polymorphism with higher levels of TC (SMD = 0.07, 95% CI = 0.01–0.14, *P* = 0.04) was only detected in healthy subjects, but not in CHD patients, T2DM patients and hyperlipidemia patients; furthermore, association of the rs662 polymorphism with higher levels of LDL-C was detected in T2DM patients and healthy subjects, but not in CHD patients and hyperlipidemia patients; continuously, association of the rs662 polymorphism with lower levels of HDL-C (SMD = − 0.06, 95% CI = − 0.12--0.01, *P* = 0.02) was only detected in CHD patients, but not in T2DM patients, hyperlipidemia patients and healthy subjects (Table 2).

### Heterogeneity analysis

In the Ox-LDL association analysis, there was significant heterogeneity in the analysis with all comparisons (*I*^*2*^ = 79.8%, *P*_*heterogeneity*_ <  0.01). Two comparisons (Lakshmy R1 2010, Zhang CH 2016) were identified as the main contributors to the heterogeneity by using Galbraith plot. The SMD value and 95% CI (SMD = 0.17, 95% CI = 0.03–0.31, *P*_*heterogeneity*_ = 0.27, *P*_*SMD*_ = 0.02) did not change substantially after excluding these outlier comparisons.

In the lipids association analysis, there was significant heterogeneity in the total comparisons for TG (*I*^*2*^ = 58.2%, *P*_*heterogeneity*_ <  0.01), TC (*I*^*2*^ = 44.5%, *P*_*heterogeneity*_ <  0.01), LDL-C (*I*^*2*^ = 55.2%, *P*_*heterogeneity*_ <  0.01) and HDL-C (*I*^*2*^ = 69.8%, *P*_*heterogeneity*_ <  0.01). Nine comparisons, 9 comparisons, 10 comparisons and 14 comparisons were respectively identified as the main contributors to the heterogeneity for TG, TC, LDL-C and HDL-C by using Galbraith plots. The SMD value and 95% CI of TG (SMD = 0.03, 95% CI = 0.00–0.06, *P*_*heterogeneity*_ = 0.87, *P*_*SMD*_ = 0.03), LDL-C (SMD = 0.02, 95% CI = 0.00–0.05, *P*_*heterogeneity*_ = 0.54, *P*_*SMD*_ = 0.05) and HDL-C (SMD = 0.01, 95% CI = − 0.02-0.04, *P*_*heterogeneity*_ = 0.09, *P*_*SMD*_ = 0.42) did not change substantially after excluding these outlier comparisons. However, The SMD values and 95% CIs of TC (SMD = 0.01, 95% CI = − 0.02-0.04, *P*_*heterogeneity*_ = 0.17, *P*_*SMD*_ = 0.54) changed significantly after excluding these outlier comparisons.

### Publication bias test

Begg’s test and Egger’s test were used to evaluate the publication bias of the included studies, and no publication bias was detected.

## Discussion

Several meta-analyses [26–29] demonstrated that the R allele of rs662 polymorphism in *PON1* is significantly associated with increased risk of CHD, but the underlying mechanisms have not yet been clarified. In the present study, we found that the R allele of the rs662 polymorphism is significantly associated with increased levels of Ox-LDL, TG, TC and LDL-C, but there is no effect of the rs662 polymorphism on HDL-C was observed. It suggests that the association between the rs662 polymorphism and CHD may partly be mediated by high Ox-LDL levels as well as abnormal lipid levels caused by the variant R allele.

Dyslipidemia is closely associated with the progression of coronary atherosclerosis, and it accounts for around 50% of the population-attributable risk of CHD [30]. According to the 2013 ACC/AHA blood cholesterol guidelines [31] and the Adult Treatment Panel III (ATP III) Guidelines [32] of the United States, LDL-C was considered as the major cause of CHD and used as the primary target for therapy, and the other lipid parameters were used as the secondary or supplementary targets. Studies have shown that Ox-LDL was an independent risk factor for CHD [33, 34], and the underlying mechanisms possibly involved in promoting foam cells formation [35] and increasing endothelial injury [36], oxidative stress [37], and vascular inflammation [38].

Several possible reasons could be proposed to explain the association between the rs662 polymorphism and circulating Ox-LDL level. At first, PON1 play a major role in hydrolyzing Ox-LDL [39, 40], and the decreased levels of PON1 activity may lead to increase the circulating levels of Ox-LDL. Secondly, the decreased levels of PON1 activity might reduce the capacity of PON1-mediated inhibition of LDL-C oxidation [41, 42], which also leads to increase levels of circulating Ox-LDL [43]. In this meta-analysis, significantly higher levels of TG, TC and LDL-C were detected in the R carriers comparing with the subjects with the QQ genotype. The mechanisms in which the rs662 polymorphism modulates plasma lipid levels have not been clarified yet. One explanation could be that the rs662 polymorphism indirectly affects plasma lipid levels through the mediation of Ox-LDL. As mentioned above, increased Ox-LDL levels in blood can cause endothelial injury, oxidative stress, and vascular inflammation. All these events are likely to trigger the development of dyslipidemia [44–47].

In most of the studies included in Ox-LDL and lipids association analysis, a dominant model was used, i.e. RR + QR vs. QQ. Therefore, a dominant model was adopted in the meta-analysis between the rs662 polymorphism and plasma levels of Ox-LDL and lipids to ensure adequate statistical power. In subgroup analysis, we found that the differences in Ox-LDL, TG, TC and LDL-C levels between the genotypes were only from Asian populations, in which the SMD values were bigger when compared to those from Caucasians, Africans and the subjects of other ethnicities (Table 2). It indicates that there is an interaction between the rs662 polymorphism and ethnicity in modulating the plasma levels of Ox-LDL and lipids. The associations of the rs662 polymorphism with Ox-LDL, TG, TC and LDL-C in Asians were in line with the finding that there was a stronger association between the rs662 polymorphism and CHD in Asians than in other ethnicities [26, 27, 48]. Studies are needed to elucidate the reasons that the rs662 polymorphism had different effects on Ox-LDL, blood lipids and CHD risk in different ethnicities.

In the Ox-LDL and lipids association analysis, subgroup analysis by gender and health status were performed since they might be important factors affecting the associations between the rs662 polymorphism and plasma levels of Ox-LDL and lipids. For example, the present meta-analysis indicated that gender might modulate the association of the rs662 polymorphism with LDL-C levels since the significant association was only existed in females, but not in males (Table 2). Health status might also modulate the associations of the rs662 polymorphism with Ox-LDL and lipids levels. The significant association of the rs662 polymorphism with TC levels was only existed in healthy subjects, but not in the patients with CHD, T2DM and hyperlipidemia (Table 2). The significant associations of the rs662 polymorphism with Ox-LDL and HDL-C levels were only existed in CHD patients, but not in T2DM patients, hyperlipidemia patients and healthy subjects (Table 2).

Several limitations of the present meta-analysis should be noted. First of all, dyslipidemia is involved in a large number of genes as well as some environmental factors. However, the interactions of the rs662 polymorphism with other polymorphic loci or environmental factors on plasma levels of Ox-LDL and lipids have not been investigated in this meta-analysis due to the lack of original data from the included studies. In other words, more precise results could have been gained if more detailed individual data were available, or the stratification analyses based on the environmental factors such as diet, exercise, smoking status, etc., were performed. Secondly, a relatively small number of subjects were included in the association analysis for Ox-LDL due to the limited number of studies that met the inclusion criteria, which may reduce the statistic power and even cause type I error (false-positive results). Thirdly, this meta-analysis only included the studies published in English and Chinese as it is very difficult to get the full papers published in various languages.

## Conclusions

The meta-analysis suggests that the rs662 polymorphism is significantly associated with abnormal levels of Ox-LDL and lipids, which may partly explain the significant association between the rs662 polymorphism and CHD.

## Additional files

Additional file 1: The reference list for the studies included in the present meta-analysis. (DOCX 26 kb)

Additional file 2: **Table S1.** Characteristics of the individual studies included in the meta-analysis of Ox-LDL levels and plasma lipid levels for the *PON1* rs662 polymorphism; **Table S2.** Ox-LDL levels by the genotypes of the *PON1* rs662 polymorphism; **Table S3.** Plasma lipid levels by the genotypes of the *PON1* rs662 polymorphism. (DOC 649 kb)

## Abbreviations

95% CI: 95% confidence interval
CHD: Coronary heart disease
HDL-C: High-density lipoprotein cholesterol
HWE: Hardy-Weinberg equilibrium
LDL-C: Low-density lipoprotein cholesterol
Ox-LDL: Oxidized low-density lipoprotein
SMD: Standardized mean difference
T2DM: Type 2 diabetes mellitus
TC: Total cholesterol
TG: Triglycerides
