# Supplementary material for: Associations of the PON1 rs662 polymorphism with circulating oxidized low-density lipoprotein and lipid levels: a systematic review and meta-analysis
Source: Lipids Health Dis. 2018 Dec 13;17:281. doi: 10.1186/s12944-018-0937-8 (PMC6293622; doi:10.1186/s12944-018-0937-8)
Supplement: Supplementary file 1 — The reference list for the studies included in the present meta-analysis. (DOCX 26 kb) [file 12944_2018_937_MOESM1_ESM.docx]

**The reference list for the studies included in the present meta-analysis**

**R1.** Ruiz J, Blanché H, James RW, Garin MC, Vaisse C, Charpentier G, Cohen N, Morabia A, Passa P, Froguel P. Gln-Arg192 polymorphism of paraoxonase and coronary heart disease in type 2 diabetes. Lancet. 1995; 346(8979): 869-72.

**R2.** Antikainen M, Murtomäki S, Syvänne M, Pahlman R, Tahvanainen E, Jauhiainen M, Frick MH, Ehnholm C. The Gln-Arg191 polymorphism of the human paraoxonase gene (HUMPONA) is not associatedwith the risk of coronary artery disease in Finns. J Clin Invest. 1996; 98(4): 883-5.

**R3.** Suehiro T, Nakauchi Y, Yamamoto M, Arii K, Itoh H, Hamashige N, Hashimoto K. Paraoxonase gene polymorphism in Japanese subjects with coronary heart disease. Int J Cardiol. 1996; 57(1): 69-73.

**R4.** Odawara M, Tachi Y, Yamashita K. Paraoxonase polymorphism (Gln192-Arg) is associated with coronary heart disease in Japanesenoninsulin-dependent diabetes mellitus. J Clin Endocrinol Metab. 1997; 82(7): 2257-60.

**R5.** Sanghera DK, Saha N, Aston CE, Kamboh MI. Genetic polymorphism of paraoxonase and the risk of coronary heart disease. Arterioscler Thromb Vasc Biol. 1997; 17(6): 1067-73.

**R6.** Pati N, Pati U. Paraoxonase gene polymorphism and coronary artery disease in Indian subjects. Int J Cardiol. 1998; 66(2): 165-8.

**R7.** Ombres D, Pannitteri G, Montali A, Candeloro A, Seccareccia F, Campagna F, Cantini R, Campa PP, Ricci G, Arca M. The gln-Arg192 polymorphism of human paraoxonase gene is not associated with coronary artery disease in italian patients. Arterioscler Thromb Vasc Biol. 1998; 18(10): 1611-6.

**R8.** Ko YL, Ko YS, Wang SM, Hsu LA, Chang CJ, Chu PH, Cheng NJ, Chen WJ, Chiang CW, Lee YS. The Gln-Arg 191 polymorphism of the human paraoxonase gene is not associated with the risk of coronary artery disease among Chinese in Taiwan. Atherosclerosis. 1998; 141(2): 259-64.

**R9.** Hasselwander O, Savage DA, McMaster D, Loughrey CM, McNamee PT, Middleton D, Nicholls DP, Maxwell AP, Young IS. Paraoxonase polymorphisms are not associated with cardiovascular risk in renal transplant recipients. Kidney Int. 1999; 56(1): 289-98.

**R10.** Singh S, Verma M, Nain CK, Leelamma CO, Goel RC. Paraoxonase (PON1) polymorphism & its relation with lipids in north west Indian Punjabis. Indian J Med Res. 1999; 110: 133-7.

**R11.** Dessì M, Gnasso A, Motti C, Pujia A, Irace C, Casciani S, Staffa F, Federici G, Cortese C. Influence of the human paraoxonase polymorphism (PON1 192) on the carotid-wall thickening in a healthy population. Coron Artery Dis. 1999; 10(8): 595-9.

**R12.** Pfohl M, Koch M, Enderle MD, Kühn R, Füllhase J, Karsch KR, Häring HU. Paraoxonase 192 Gln/Arg gene polymorphism, coronary artery disease, and myocardial infarctionin type 2 diabetes. Diabetes. 1999; 48(3): 623-7.

**R13.** MacKness B, Mackness MI, Durrington PN, Arrol S, Evans AE, McMaster D, Ferrières J, Ruidavets JB, Williams NR, Howard AN. Paraoxonase activity in two healthy populations with differing rates of coronary heart disease. Eur J Clin Invest. 2000; 30(1): 4-10.

**R14.** Aubó C, Sentí M, Marrugat J, Tomás M, Vila J, Sala J, Masiá R. Risk of myocardial infarction associated with Gln/Arg 192 polymorphism in the human paraoxonase gene and diabetes mellitus. The REGICOR Investigators. Eur Heart J. 2000; 21(1): 33-8.

**R15.** Imai Y, Morita H, Kurihara H, Sugiyama T, Kato N, Ebihara A, Hamada C, Kurihara Y, Shindo T, Oh-hashi Y, Yazaki Y. Evidence for association between paraoxonase gene polymorphisms and atherosclerotic diseases. Atherosclerosis. 2000; 149(2): 435-42.

**R16.** Gardemann A, Philipp M, Hess K, Katz N, Tillmanns H, Haberbosch W. The paraoxonase Leu-Met54 and Gln-Arg191 gene polymorphisms are not associated with the risk of coronary heart disease. Atherosclerosis. 2000; 152(2): 421-31.

**R17.** Sen-Banerjee S, Siles X, Campos H. Tobacco smoking modifies association between Gln-Arg192 polymorphism of human paraoxonase gene and risk of myocardial infarction. Arterioscler Thromb Vasc Biol. 2000; 20(9): 2120-6.

**R18.** Turban S, Fuentes F, Ferlic L, Brugada R, Gotto AM, Ballantyne CM, Marian AJ. A prospective study of paraoxonase gene Q/R192 polymorphism and severity, progression andregression of coronary atherosclerosis, plasma lipid levels, clinical events and response to fluvastatin. Atherosclerosis. 2001; 154(3): 633-40.

**R19.** Liu R, Bai H, Deng JL, Liu Y, Huang MH, Li X, Liu BW. The Paraoxonase Gln-Arg 192 Polymorphism in Patients with Coronary Heart Disease in Chinese Population. JWCUMS. 2001; 32(3): 385-388.

**R20.** Tomás M, Sentí M, Elosua R, Vila J, Sala J, Masià R, Marrugat J. Interaction between the Gln-Arg 192 variants of the paraoxonase gene and oleic acid intake as a determinant of high-density lipoprotein cholesterol and paraoxonase activity. Eur J Pharmacol. 2001; 432(2-3): 121-8.

**R21.** Koch M, Hering S, Barth C, Ehren M, Enderle MD, Pfohl M. Paraoxonase 1 192 Gln/Arg gene polymorphism and cerebrovascular disease: interaction with type 2 diabetes. Exp Clin Endocrinol Diabetes. 2001; 109(3): 141-5.

**R22.** Yamada A, Shoji T, Tahara H, Emoto M, Nishizawa Y. Effect of insulin resistance on serum paraoxonase activity in a nondiabetic population. Metabolism. 2001; 50(7): 805-11.

**R23.** Liu R, Bai H, Liu Y, Huang MH, Fan P, Liu BW. The Paraoxonase Gln-Arg 192 Polymorphism in Patients with Endogenous Hypertriglyceridemia in Chinese Population. JWCUMS. 2002; 33(2): 229-232.

**R24.** Kuremoto K, Watanabe Y, Ohmura H, Shimada K, Mokuno H, Daida H. R/R genotype of human paraoxonase (PON1) is more protective against lipoprotein oxidation and coronary artery disease in Japanese subjects. J Atheroscler Thromb. 2003; 10(2): 85-92.

**R25.** Qian QW, Qian SH, Wang SJ. Association of paraoxonase 1 arylersterase gene polymorphism in patients with type 2 diabetes and coronary heart disease. J Clin Cardiol (China). 2003; 19(10): 606-609.

**R26.** Qian SH, Qian QW. Research of Arylesterase activity and its 192 polymorphism in patients with uremia patients with diabetic nephropathy. Chin J Nephrol. 2003; 19(3): 177-178.

**R27.** Robertson KS, Hawe E, Miller GJ, Talmud PJ, Humphries SE; Northwick Park Heart Study II. Human paraoxonase gene cluster polymorphisms as predictors of coronary heart disease risk in the prospective Northwick Park Heart Study II. Biochim Biophys Acta. 2003; 1639(3): 203-12.

**R28.** Ueno T, Shimazaki E, Matsumoto T, Watanabe H, Tsunemi A, Takahashi Y, Mori M, Hamano R, Fujioka T, Soma M, Matsumoto K, Kanmatsuse K. Paraoxonase 1 polymorphism Leu-Met55 is associated with cerebral infarction in Japanese population. Med Sci Monit. 2003; 9(6): CR208-12.

**R29.** Hu Y, Tian H, Liu R. Gln-Arg192 polymorphism of paraoxonase 1 is associated with carotid intima-media thickness inpatients of type 2 diabetes mellitus of Chinese. Diabetes Res Clin Pract. 2003; 61(1): 21-7.

**R30.** Campo S, Sardo MA, Trimarchi G, Bonaiuto M, Fontana L, Castaldo M, Bonaiuto A, Saitta C, Bitto A, Manduca B, Riggio S, Saitta A. Association between serum paraoxonase (PON1) gene promoter T(-107)C polymorphism, PON1 activity and HDL levels in healthy Sicilian octogenarians. Exp Gerontol. 2004; 39(7): 1089-94.

**R31.** Xiao ZJ, Zhao SP, Nie S, Tan LM, Jiang B, Wu J, Zhou HN, Li H. The relationship between the gene polymorphism in paranoxonase 1 and cerebral hemorrhage. J Apoplexy and Nervous Diseases. 2004; 21(4): 320-322.

**R32.** Agachan B, Yilmaz H, Karaali Z, Isbir T. Paraoxonase 55 and 192 polymorphism and its relationship to serum paraoxonase activity and serum lipids in Turkish patients with non-insulin dependent diabetes mellitus. Cell Biochem Funct. 2004; 22(3): 163-8.

**R33.** Srinivasan SR, Li S, Chen W, Tang R, Bond MG, Boerwinkle E, Berenson GS. Q192R polymorphism of the paraoxanase 1 gene and its association with serum lipoproteinvariables and carotid artery intima-media thickness in young adults from a biracial community. The Bogalusa Heart Study. Atherosclerosis. 2004; 177(1): 167-74.

**R34.** Li SY, Li JY, Wei G, Ji QD, Wang PX. Study on the Relationship between Paraoxonase-1 (PON-1) and Type 2 Diabetes Mell itus Complicated by Coronary Artery Disease. Tian jin Med J. 2004; 32(5): 273-276.

**R35.** Oliveira SA, Mansur AP, Ribeiro CC, Ramires JA, Annichino-Bizzacchi JM. PON1 M/L55 mutation protects high-risk patients against coronary artery disease. Int J Cardiol. 2004; 94(1): 73-7.

**R36.** Xiao ZJ, Zhao SP, Nie S, Tan LM, Wu J, Jiang B, Lu W. Effect of the interaction between paranoxonase 1 and ATP-binding cassette transporter 1 gene polymorphism on serum lipid level. Zhonghua Yi Xue Yi Chuan Xue Za Zhi. 2005; 22(3): 272-6.

**R37.** Blatter Garin MC, Moren X, James RW. Paraoxonase-1 and serum concentrations of HDL-cholesterol and apoA-I. J Lipid Res. 2006; 47(3): 515-20.

**R38.** Qian SH, Qian QW. Research of Arylesterase/paraoxonase 1 activity and its 192 polymorphism in patients with kidney- transplanted. China Journal of Modern Medicine. 2006; 16(16): 2444-2452.

**R39.** Aydin M, Gencer M, Cetinkaya Y, Ozkok E, Ozbek Z, Kilic G, Orken C, Tireli H, Kara I. PON1 55/192 polymorphism, oxidative stress, type, prognosis and severity of stroke. IUBMB Life. 2006; 58(3): 165-72.

**R40.** Manresa JM, Zamora A, Tomás M, Sentí M, Fitó M, Covas MI, Alcántara M, Latorre G, Escurriol V, Domingues S, Marrugat J. Relationship of classical and non-classical risk factors with genetic variants relevant to coronary heart disease. Eur J Cardiovasc Prev Rehabil. 2006; 13(5): 738-44.

**R41.** Huang Q, Liu YH, Yang QD, Xiao B, Ge L, Zhang N, Xia J, Zhang L, Liu ZJ. Human serum paraoxonase gene polymorphisms, Q192R and L55M, are not associated with the risk of cerebral infarction in Chinese Han population. Neurol Res. 2006; 28(5): 549-54.

**R42.** Juretić D, Motejlkova A, Kunović B, Rekić B, Flegar-Mestrić Z, Vujić L, Mesić R, Lukac-Bajalo J, Simeon-Rudolf V. Paraoxonase/arylesterase in serum of patients with type II diabetes mellitus. Acta Pharm. 2006; 56(1): 59-68.

**R43.** Rios DL, D'Onofrio LO, Cerqueira CC, Bonfim-Silva R, Carvalho HG, Santos-Filho A, Galvão-Castro B. Paraoxonase 1 gene polymorphisms in angiographically assessed coronary artery disease:evidence for gender interaction among Brazilians. Clin Chem Lab Med. 2007; 45(7): 874-8.

**R44.** Lahiry P, Ban MR, Pollex RL, Feldman RD, Sawyez CG, Huff MW, Young TK, Bjerregaard P, Hegele RA. Common variants APOC3, APOA5, APOE and PON1 are associated with variation in plasmalipoprotein traits in Greenlanders. Int J Circumpolar Health. 2007; 66(5): 390-400.

**R45.** Irace C, Cortese C, Fiaschi E, Scavelli F, Liberatoscioli L, Federici G, Gnasso A. The influence of PON1 192 polymorphism on endothelial function in diabetic subjects with or without hypertension. Hypertens Res. 2008; 31(3): 507-13.

**R46.** Fu R, Sun YM, Su Y, Wu Y, Luan Y. Effect of statin therapy on plasma high-density lipoprotein-cholesterol levels is modified by paraoxonase 1 in Chinese patients with coronary heart disease. Clin Exp Pharmacol Physiol. 2008; 35(8): 982-3.

**R47.** Garcés C, López-Simón L, Rubio R, Benavente M, Cano B, Ortega H, de Oya M. High-density lipoprotein cholesterol and paraoxonase 1 (PON1) genetics and serum PON1 activity in prepubertal children in Spain. Clin Chem Lab Med. 2008; 46(6): 809-13.

**R48.** Guxens M, Tomás M, Elosua R, Aldasoro E, Segura A, Fiol M, Sala J, Vila J, Fullana M, Sentí M, Vega G, de la Rica M, Marrugat J; investigadores del estudio IBERICA. [Association between paraoxonase-1 and paraoxonase-2 polymorphisms and the risk of acute myocardial infarction]. Rev Esp Cardiol. 2008; 61(3): 269-75.

**R49.** van Himbergen TM, van der Schouw YT, Voorbij HA, van Tits LJ, Stalenhoef AF, Peeters PH, Roest M. Paraoxonase (PON1) and the risk for coronary heart disease and myocardial infarction in ageneral population of Dutch women. Atherosclerosis. 2008; 199(2): 408-14.

**R50.** van den Berg SW, Jansen EH, Kruijshoop M, Beekhof PK, Blaak E, van der Kallen CJ, van Greevenbroek MM, Feskens EJ. Paraoxonase 1 phenotype distribution and activity differs in subjects with newly diagnosed Type 2 diabetes (the CODAM Study). Diabet Med. 2008; 25(2): 186-93.

**R51.** Regieli JJ, Jukema JW, Doevendans PA, Zwinderman AH, Kastelein JJ, Grobbee DE, van der Graaf Y. Paraoxonase variants relate to 10-year risk in coronary artery disease: impact of a high-density lipoprotein-bound antioxidant in secondary prevention. J Am Coll Cardiol. 2009; 54(14): 1238-45.

**R52.** Porntadavity S, Tantrarongroj S, Pidetcha P, Dansethakul P, Suwannathon L. Paraoxonasel phenotype distribution in Thais. J Med Assoc Thai. 2009; 92(3): 405-12.

**R53.** Birjmohun RS, Vergeer M, Stroes ES, Sandhu MS, Ricketts SL, Tanck MW, Wareham NJ, Jukema JW, Kastelein JJ, Khaw KT, Boekholdt SM. Both paraoxonase-1 genotype and activity do not predict the risk of future coronary artery disease; the EPIC-Norfolk Prospective Population Study. PLoS One. 2009; 4(8): e6809.

**R54.** Aydin M, Gokkusu C, Ozkok E, Tulubas F, Unlucerci Y, Pamukcu B, Ozbek Z, Umman B. Association of genetic variants in Methylenetetrahydrofolate Reductase and Paraoxonase-1 genes with homocysteine, folate and vitamin B12 in coronary artery disease. Mol Cell Biochem. 2009; 325(1-2): 199-208.

**R55.** Gluba A, Pietrucha T, Banach M, Piotrowski G, Rysz J. The role of polymorphisms within paraoxonases (192 Gln/Arg in PON1 and 311Ser/Cys in PON2) in the modulation of cardiovascular risk: a pilot study. Angiology. 2010; 61(2): 157-65.

**R56.** Mohamed RH, Mohamed RH, Karam RA, Abd El-Aziz TA. The relationship between paraoxonase1-192 polymorphism and activity with coronary artery disease. Clin Biochem. 2010; 43(6): 553-8.

**R57.** Likidlilid A, Akrawinthawong K, Poldee S, Sriratanasathavorn C. Paraoxonase 1 polymorphisms as the risk factor of coronary heart disease in a Thai population. Acta Cardiol. 2010; 65(6): 681-91.

**R58.** Chen XJ, Pan SZ, Zen J. Association of PON1 and PON2 gene polymorphisms with plasma Ox-LDL level in type 2 diabetic patients. Chin J Diabetes. 2010; 18(4): 264-266.

**R59.** Lakshmy R, Ahmad D, Abraham RA, Sharma M, Vemparala K, Das S, Reddy KS, Prabhakaran D. Paraoxonase gene Q192R & L55M polymorphisms in Indians with acute myocardial infarction &association with oxidized low density lipoprotein. Indian J Med Res. 2010; 131: 522-9.

**R60.** Altuner D, Ates I, Suzen SH, Koc GV, Aral Y, Karakaya A. The relationship of PON1 QR 192 and LM 55 polymorphisms with serum paraoxonase activities of Turkish diabetic patients. Toxicol Ind Health. 2011; 27(10): 873-8.

**R61.** Trenk D, Hochholzer W, Fromm MF, Zolk O, Valina CM, Stratz C, Neumann FJ. Paraoxonase-1 Q192R polymorphism and antiplatelet effects of clopidogrel in patients undergoing elective coronary stent placement. Circ Cardiovasc Genet. 2011; 4(4): 429-36.

**R62.** Vaisi-Raygani A, Ghaneialvar H, Rahimi Z, Tavilani H, Pourmotabbed T, Shakiba E, Vaisi-Raygani A, Kiani A, Aminian M, Alibakhshi R, Bartels C. Paraoxonase Arg 192 allele is an independent risk factor for three-vessel stenosis of coronary artery disease. Mol Biol Rep. 2011; 38(8): 5421-8.

**R63.** Sibbing D, Koch W, Massberg S, Byrne RA, Mehilli J, Schulz S, Mayer K, Bernlochner I, Schömig A, Kastrati A. No association of paraoxonase-1 Q192R genotypes with platelet response to clopidogrel and risk of stent thrombosis after coronary stenting. Eur Heart J. 2011; 32(13): 1605-13.

**R64.** Wang Y, Liu H, Fan P, Bai H, Zhang J, Zhang F. Evidence for association between paraoxonase 1 gene polymorphisms and polycystic ovarian syndrome in southwest Chinese women. Eur J Endocrinol. 2012; 166(5): 877-85.

**R65.** Haj Mouhamed D, Ezzaher A, Mechri A, Neffati F, Omezzine A, Bouslama A, Gaha L, Douki W, Najjar MF. Effect of cigarette smoking on paraoxonase 1 activity according to PON1 L55M and PON1 Q192R gene polymorphisms. Environ Health Prev Med. 2012; 17(4): 316-21.

**R66.** Moura LM, Faria S, Brito M, Pinto FJ, Kristensen SD, Barros IM, Rajamannan N, Rocha-Gonçalves F. Relationship of PON1 192 and 55 gene polymorphisms to calcific valvular aortic stenosis. Am J Cardiovasc Dis. 2012; 2(2): 123-32.

**R67.** Yang J, Zhou JS, Tan J, He BS, Zou JJ. Paraoxonase-1 Q192R polymorphism is not associated with clopidogrel response in Chinese stroke patients. Pharmazie. 2012; 67(12): 1026-9.

**R68.** Murugan MM, Alagirisamy R, Manokaran S, Balakrishnan A, Sundaresen V. THE ROLE OF PARAOXONASE ACTIVITY AND (Q/R192) POLYMORPHISM IN CORONARY ARTERY DISEASE. Int. J. Int sci. Inn. Tech. Sec. B. 2012; 1(3): 34-40.

**R69.** Kucuk ST, Ademoglu E, Turkoglu UM, Bilge AK. Distribution of PON L/M55 and Q/R192 Genotypes in Turkish Patients with Angiographically-Defined Coronary Artery Disease: Effects on Serum Lipids. Turkiye Klinikleri Journal of Medical Sciences. 2013; 33(3): 769-776.

**R70.** Hassan MA, Al-Attas OS, Hussain T, Al-Daghri NM, Alokail MS, Mohammed AK, Vinodson B. The Q192R polymorphism of the paraoxonase 1 gene is a risk factor for coronary artery diseasein Saudi subjects. Mol Cell Biochem. 2013; 380(1-2): 121-8.

**R71.** Fekih O, Triki S, Hellara I, Neffati F, Rejeb J, Ommezzine A, Chouchane S, Guediche MN, Bouslama A, Najjar MF. Can paraoxonase 1 polymorphisms (L55 M and Q192 R) protect children with type 1 diabetes against lipid abnormalities? J Clin Lipidol. 2014; 8(3): 249-55.

**R72.** Shao ZY, Li JR, Wang XD. Analysis of paraoxonase 1 gene polymorphisms in type 2 diabetic patients with coronary arterydisease. Chin J Cardiovasc Med. 2014; 19(6): 426-429.

**R73.** Bortolasci CC, Vargas HO, Souza-Nogueira A, Barbosa DS, Moreira EG, Nunes SO, Berk M, Dodd S, Maes M. Lowered plasma paraoxonase (PON)1 activity is a trait marker of major depression and PON1 Q192R gene polymorphism-smoking interactions differentially predict the odds of major depression and bipolar disorder. J Affect Disord. 2014; 159: 23-30.

**R74.** Macharia M, Kengne AP, Blackhurst DM, Erasmus RT, Matsha TE. Paraoxonase 1 genetic polymorphisms in a mixed ancestry African population. Mediators Inflamm. 2014; 2014: 217019.

**R75.** Bounafaa A, Berrougui H, Ghalim N, Nasser B, Bagri A, Moujahid A, Ikhlef S, Camponova P, Yamoul N, Simo OK, Essamadi A, Khalil A. Association between Paraoxonase 1 (PON1) Polymorphisms and the Risk of Acute Coronary Syndrome in a North African Population. PLoS One. 2015; 10(8): e0133719.

**R76.** Alegría-Torres JA, García-Domínguez ML, Cruz M, Aradillas-García C. Q192R polymorphism of paraoxonase 1 gene associated with insulin resistance in Mexican children. Arch Med Res. 2015; 46(1): 78-83.

**R77.** Scherrer DZ, Zago VH, Vieira IC, Parra ES, Panzoldo NB, Alexandre F, Secolin R, Baracat J, Quintão EC, Faria EC. p.Q192R SNP of PON1 seems not to be Associated with Carotid Atherosclerosis Risk Factors in an Asymptomatic and Normolipidemic Brazilian Population Sample. Arq Bras Cardiol. 2015; 105(1): 45-52.

**R78.** Kolesnikova LI, Bairova TA, Pervushina OA, Grebenkina LA. [Association of (192) Q>R polymorphism of the paraoxonase gene with a lipid profile and components of lipid peroxidation and antioxidant protection in populations of Russians and Buryats from Eastern Siberia]. Genetika. 2015; 51(2): 236-41.

**R79.** Liang X, Zhang ZY, Liang GY, Li Y, Jiang MY, Xia TL, Wei P, Tan J. Association between PON1 genes polymorphism with the serum lipid levels of Guangxi longevity population in Bama county. Zhong Guo Lao Nian Xue Za Zhi. 2016; 36: 1072-1074.

**R80.** Zhang CH, Chen XJ. The correlation between gene polymorphism of paraoxonase1 and patients with acute myocardial infarction. Journal of Ningxia Medical University. 2016; 38(3): 304-306.

**R81.** Szpakowicz A, Pepinski W, Waszkiewicz E, Maciorkowska D, Skawronska M, Niemcunowicz-Janica A, Dobrzycki S, Musial WJ, Kaminski KA. The influence of renal function on the association of rs854560 polymorphism of paraoxonase 1 gene with long-term prognosis in patients after myocardial infarction. Heart Vessels. 2016; 31(1): 15-22.

**R82.** Fridman O, Gariglio L, Riviere S, Porcile R, Fuchs A, Potenzoni M. Paraoxonase 1 gene polymorphisms and enzyme activities in coronary artery disease and itsrelationship to serum lipids and glycemia. Arch Cardiol Mex. 2016; 86(4): 350-357.

**R83.** Zargari M, Sharafeddin F, Mahrooz A, Alizadeh A, Masoumi P. The common variant Q192R at the paraoxonase 1 (PON1) gene and its activity are responsible for a portion of the altered antioxidant status in type 2 diabetes. Exp Biol Med (Maywood). 2016; 241(14): 1489-96.

**R84.** Alharbi KK, Alharbi FK, Ghneim HK, Al-Sulaiman AM, Alodhayani AA, Tabassum SN, Khan IA. Amendment of amino acid in Q192R genetic polymorphism of paraoxonase 1 is a conventional risk factor for type 2 diabetes mellitus in the Saudi population. Int J Clin Exp Med. 2016; 9(8): 16605-16612.

**R85.** Martínez-Quintana E, Rodríguez-González F, Medina-Gil JM, Garay-Sánchez P, Tugores A. Paraoxonase 1 (Q192R) gene polymorphism, coronary heart disease and the risk of a new acutecoronary event. Clin Investig Arterioscler. 2017; 29(1): 1-6.
