# Supplementary material for: Associations of the PON1 rs662 polymorphism with circulating oxidized low-density lipoprotein and lipid levels: a systematic review and meta-analysis
Source: Lipids Health Dis. 2018 Dec 13;17:281. doi: 10.1186/s12944-018-0937-8 (PMC6293622; doi:10.1186/s12944-018-0937-8)
Supplement: Supplementary file 2 — Table S1. Characteristics of the individual studies included in the meta-analysis of Ox-LDL levels and plasma lipid levels for the PON1 rs662 polymorphism; Table S2. Ox-LDL levels by the genotypes of the PON1 rs662 polymorphism; Table S3. Plasma lipid levels by the genotypes of the PON1 rs662 polymorphism. (DOC 649 kb) [file 12944_2018_937_MOESM2_ESM.doc]

**Table S1.** Characteristics of the individual studies included in the meta-analysis of Ox-LDL levels and plasma lipid levels for the *PON1* rs662 polymorphism

**Table S2.** Ox-LDL levels by the genotypes of the *PON1* rs662 polymorphism.

**Table S3:** Plasma lipid levels by the genotypes of the *PON1* rs662 polymorphism

**Table S1 : Characteristics of the individual studies included in the meta-analysis of Ox-LDL levels and plasma lipid levels for the *PON1* rs662 polymorphism**

| **First author, reference** | **year** | **Ethnicity** | **Gender** | **Study population** | **Outcomes** |
| --- | --- | --- | --- | --- | --- |
| Ruiz J [R1] | 1995 | Caucasian | M/F | T2DM patients | TG, TC, HDL-C |
| Antikainen M [R2] | 1996 | Caucasian | M/F | CHD patients | TG, TC, LDL-C, HDL-C |
| Suehiro T [R3] | 1996 | Asian | M/F | CHD patients | TG, TC, LDL-C, HDL-C |
| Odawara M [R4] | 1997 | Asian | M/F | T2DM patients | TG, TC, HDL-C |
| Sanghera DK1 [R5] | 1997 | Other ethnic | M/F | Control subjects | TG, TC, LDL-C, HDL-C |
| Sanghera DK2 [R5] | 1997 | Asian | M/F | Control subjects | TG, TC, LDL-C, HDL-C |
| Pati N [R6] | 1998 | Caucasian | M/F | CHD patients | HDL-C |
| Ombres D [R7] | 1998 | Caucasian | M/F | CHD patients and control subjects | TG, TC, LDL-C, HDL-C |
| Ko YL [R8] | 1998 | Asian | M/F | Control subjects | TG, TC, LDL-C, HDL-C |
| Hasselwander O [R9] | 1999 | Caucasian | M/F | Renal transplant recipients | TC, LDL-C, HDL-C |
| Singh S [R10] | 1999 | Caucasian | M/F | Healthy subjects | TG, TC, LDL-C, HDL-C |
| Dessi M [R11] | 1999 | Caucasian | M/F | Healthy subjects | TG, TC, LDL-C, HDL-C |
| Pfohl M [R12] | 1999 | Caucasian | M/F | T2DM patients | TC, LDL-C, HDL-C |
| MacKness B1 [R13] | 2000 | Caucasian | M/F | Healthy subjects | TC, LDL-C, HDL-C |
| MacKness B2 [R13] | 2000 | Caucasian | M/F | Healthy subjects | TC, LDL-C, HDL-C |
| Aubó C [R14] | 2000 | Caucasian | M/F | CHD patients and control subjects | TC, LDL-C, HDL-C |
| Imai Y [R15] | 2000 | Asian | M/F | Control subjects | TG, TC, LDL-C, HDL-C |
| Gardemann A [R16] | 2000 | Caucasian | M | CHD patients and control subjects | TG, TC |
| Sen-Banerjee S1 [R17] | 2000 | Caucasian | M/F | CHD patients | TG, TC, HDL-C |
| Sen-Banerjee S2 [R17] | 2000 | Caucasian | M/F | Control subjects | TG, TC, HDL-C |
| Turban S1 [R18] | 2001 | Caucasian | M/F | CHD patients | TG, TC, LDL-C, HDL-C |
| Turban S2 [R18] | 2001 | Caucasian | M/F | CHD patients | TG, TC, LDL-C, HDL-C |
| Liu R1 [R19] | 2001 | Asian | M/F | CHD patients | TG, TC, LDL-C, HDL-C |
| Liu R2 [R19] | 2001 | Asian | M/F | Control subjects | TG, TC, LDL-C, HDL-C |
| Tomás M [R20] | 2001 | Caucasian | M | Healthy subjects | TC, LDL-C, HDL-C |
| Koch M [R21] | 2001 | Caucasian | M/F | CVD patients | TC, LDL-C, HDL-C |
| Yamada A [R22] | 2001 | Asian | M/F | Healthy subjects | TG, TC, HDL-C |
| Liu R1 [R23] | 2002 | Asian | M/F | Hyperlipidemia patients | TG, TC, LDL-C, HDL-C |
| Liu R2 [R23] | 2002 | Asian | M/F | Control subjects | TG, TC, LDL-C, HDL-C |
| Kuremoto K [R24] | 2003 | Asian | M/F | CHD patients and control subjects | TG, TC, HDL-C |
| Qian QW1 [R25] | 2003 | Asian | M/F | T2DM patients | TG, TC, LDL-C, HDL-C, Ox-LDL |
| Qian QW2 [R25] | 2003 | Asian | M/F | T2DM and CHD patients | TG, TC, LDL-C, HDL-C, Ox-LDL |
| Qian QW3 [R25] | 2003 | Asian | M/F | CHD patients | TG, TC, LDL-C, HDL-C, Ox-LDL |
| Qian QW4 [R25] | 2003 | Asian | M/F | Control subjects | TG, TC, LDL-C, HDL-C, Ox-LDL |
| Qian SH1 [R26] | 2003 | Asian | M/F | Primary hyperlipoproteinemia patients | TG, TC, LDL-C, HDL-C, Ox-LDL |
| Qian SH2 [R26] | 2003 | Asian | M/F | DN caused Uremia patients | TG, TC, LDL-C, HDL-C, Ox-LDL |
| Qian SH3 [R26] | 2003 | Asian | M/F | Control subjects | TG, TC, LDL-C, HDL-C, Ox-LDL |
| Robertson KS [R27] | 2003 | Caucasian | M | Healthy subjects | HDL-C |
| Ueno T [R28] | 2003 | Asian | M/F | Cerebral infarction patients and control subjects | TG, TC, HDL-C |
| Hu Y [R29] | 2003 | Asian | M/F | T2DM patients | TG, TC, LDL-C, HDL-C |
| Campo S [R30] | 2004 | Caucasian | M/F | Healthy subjects | HDL-C |
| Xiao ZJ [R31] | 2004 | Asian | M/F | Cerebral hemorrhage patients and control subjects | TG, TC, LDL-C, HDL-C |
| Agachan B1 [R32] | 2004 | Other ethnic | M/F | Noninsulin dependent diabetes mellitus patients | TG, TC, LDL-C, HDL-C |
| Agachan B2 [R32] | 2004 | Other ethnic | M/F | Control subjects | TG, TC, LDL-C, HDL-C |
| Srinivasan SR1 [R33] | 2004 | Caucasian | M/F | Healthy subjects | TG, LDL-C, HDL-C |
| Srinivasan SR2 [R33] | 2004 | African | M/F | Healthy subjects | TG, LDL-C, HDL-C |
| Li SY [R34] | 2004 | Asian | M/F | CHD/T2DM patients and control subjects | TG, TC |
| Oliveira SA [R35] | 2004 | Other ethnic | M/F | CHD patients and control subjects | TG, TC, LDL-C, HDL-C |
| Xiao ZJ [R36] | 2005 | Asian | M/F | Stroke patients and control subjects | TG, TC, LDL-C, HDL-C |
| Blatter Garin MC1 [R37] | 2005 | Caucasian | M/F | CHD patients | LDL-C, HDL-C |
| Blatter Garin MC2 [R37] | 2005 | Caucasian | M/F | Control subjects | LDL-C, HDL-C |
| Qian SH1 [R38] | 2006 | Asian | M/F | Kidney transplant patients | TG, TC, LDL-C, HDL-C, Ox-LDL |
| Qian SH2 [R38] | 2006 | Asian | M/F | Uremia patients | TG, TC, LDL-C, HDL-C, Ox-LDL |
| Qian SH3 [R38] | 2006 | Asian | M/F | Control subjects | TG, TC, LDL-C, HDL-C, Ox-LDL |
| Aydin M1 [R39] | 2006 | Other ethnic | M/F | Stroke patients | TG, TC, LDL-C, HDL-C |
| Aydin M2 [R39] | 2006 | Other ethnic | M/F | Control subjects | TG, TC, LDL-C, HDL-C |
| Manresa JM [40] | 2006 | Caucasian | M/F | Healthy subjects | TC, LDL-C, HDL-C |
| Huang Q [R41] | 2006 | Asian | M/F | Healthy subjects | TG, TC, LDL-C, HDL-C |
| Juretić D1 [R42] | 2006 | Caucasian | M | T2DM patients | TG, TC, LDL-C, HDL-C |
| Juretić D2 [R42] | 2006 | Caucasian | F | T2DM patients | TG, TC, LDL-C, HDL-C |
| Juretić D3 [R42] | 2006 | Caucasian | M | Control subjects | TG, TC, LDL-C, HDL-C |
| Juretić D4 [R42] | 2006 | Caucasian | F | Control subjects | TG, TC, LDL-C, HDL-C |
| Rios DL1 [R43] | 2007 | Other ethnic | M | CHD patients and control subjects | TG, HDL-C |
| Rios DL2 [R43] | 2007 | Other ethnic | F | CHD patients and control subjects | TG, HDL-C |
| Rios DL3 [R43] | 2007 | African | M | CHD patients and control subjects | TG, HDL-C |
| Rios DL4 [R43] | 2007 | African | F | CHD patients and control subjects | TG, HDL-C |
| Lahiry P [R44] | 2007 | Other ethnic | M/F | Healthy subjects | TC, LDL-C |
| Irace C1 [R45] | 2008 | Caucasian | M/F | T2DM patients | TG, TC, HDL-C |
| Irace C2 [R45] | 2008 | Caucasian | M/F | Control subjects | TG, TC, HDL-C |
| Irace C3 [R45] | 2008 | Caucasian | M/F | T2DM patients | TG, TC, HDL-C |
| Irace C4 [R45] | 2008 | Caucasian | M/F | T2DM and hypertensive patients | TG, TC, HDL-C |
| Fu R [R46] | 2008 | Asian | M/F | CHD patients | TG, TC, LDL-C, HDL-C |
| Garcés C [R47] | 2008 | Caucasian | M/F | Healthy subjects | TG, TC, LDL-C, HDL-C |
| Guxens M [R48] | 2008 | Caucasian | M/F | CHD patients and control subjects | TC, LDL-C, HDL-C |
| van Himbergen TM [R49] | 2008 | Caucasian | F | Healthy subjects | HDL-C |
| van den Berg SW [R50] | 2008 | Caucasian | M/F | T2DM patients and control subjects | TC, LDL-C, HDL-C |
| Regieli JJ [R51] | 2009 | Caucasian | M | CHD patients | TC, LDL-C, HDL-C |
| Porntadavity S [R52] | 2009 | Other ethnic | M/F | Healthy subjects | TG, TC, LDL-C, HDL-C |
| Birjmohun RS [R53] | 2009 | Caucasian | M/F | CHD patients and control subjects | LDL-C, HDL-C |
| Aydin M [54] | 2009 | Other ethnic | M/F | CHD patients | TG, TC, LDL-C, HDL-C |
| Gluba A [R55] | 2010 | Caucasian | M/F | CHD patients and control subjects | TC, LDL-C, HDL-C |
| Mohamed RH [R56] | 2010 | Caucasian | M/F | CHD patients and control subjects | TG, TC, LDL-C, HDL-C |
| Likidlilid A1 [R57] | 2010 | Other ethnic | M/F | Hyperlipidemia patients | TG, TC, LDL-C, HDL-C |
| Likidlilid A2 [R57] | 2010 | Other ethnic | M/F | Control subjects | TG, TC, LDL-C, HDL-C |
| Chen XJ [R58] | 2010 | Asian | M/F | T2DM patients | TC, LDL-C, HDL-C |
| Lakshmy R1 [R59] | 2010 | Caucasian | M/F | CHD patients | HDL-C, Ox-LDL |
| Lakshmy R2 [R59] | 2010 | Caucasian | M/F | Control subjects | HDL-C, Ox-LDL |
| Altuner D1 [R60] | 2011 | Other ethnic | M/F | T2DM patients | TG, TC, LDL-C, HDL-C |
| Altuner D2 [R60] | 2011 | Other ethnic | M/F | Control subjects | TG, TC, LDL-C, HDL-C |
| Trenk D [R61] | 2011 | Caucasian | M/F | CHD patients | TC |
| Vaisi-Raygani A1 [R62] | 2011 | Caucasian | M/F | CHD patients | TG, TC, LDL-C, HDL-C |
| Vaisi-Raygani A2 [R62] | 2011 | Caucasian | M/F | Control subjects | TG, TC, LDL-C, HDL-C |
| Sibbing D [R63] | 2011 | Caucasian | M/F | Percutaneous coronary intervention patients | HDL-C, LDL-C |
| Wang Y1 [R64] | 2012 | Asian | F | Polycystic ovary syndrome patients | TG, TC, LDL-C, HDL-C |
| Wang Y2 [R64] | 2012 | Asian | F | Control subjects | TG, TC, LDL-C, HDL-C |
| Haj Mouhamed D1 [R65] | 2012 | African | M/F | Healthy subjects | HDL-C |
| Haj Mouhamed D2 [R65] | 2012 | African | M/F | Healthy subjects | HDL-C |
| Moura LM [R66] | 2012 | Caucasian | M/F | Calcific aortic valve stenosis patients and control subjects | TC, LDL-C, HDL-C |
| Yang J [R67] | 2012 | Asian | M/F | Stroke patients | LDL-C, HDL-C |
| Murugan MM1 [R68] | 2012 | Other ethnic | M/F | CHD patients | TG, TC, LDL-C, HDL-C |
| Murugan MM2 [R68] | 2012 | Other ethnic | M/F | Control subjects | TG, TC, LDL-C, HDL-C |
| Kucuk ST1 [R69] | 2013 | Other ethnic | M/F | CHD patients | TG, TC, LDL-C, HDL-C |
| Kucuk ST2 [R69] | 2013 | Other ethnic | M/F | Control subjects | TG, TC, LDL-C, HDL-C |
| Hassan MA [R70] | 2013 | Caucasian | M/F | CHD patients and control subjects | TG, TC, LDL-C, HDL-C |
| Fekih O1 [R71] | 2014 | African | M/F | TIDM patients | TG, TC, LDL-C, HDL-C |
| Fekih O2 [R71] | 2014 | African | M/F | Control subjects | TG, TC, LDL-C, HDL-C |
| Shao ZY [R72] | 2014 | Asian | M/F | DM-CHD/DM patients and control subjects | TG, TC, LDL-C, HDL-C |
| Bortolasci CC [R73] | 2014 | Other ethnic | M/F | Major depressed/Bipolar patients patients and control subjects | HDL-C |
| Macharia M [R74] | 2014 | African | M/F | T2DM/Hypertention patients and control subjects | TG, TC, LDL-C, HDL-C |
| Bounafaa A [R75] | 2015 | Caucasian | M/F | CHD patients | TG, TC |
| Alegría-Torres JA [R76] | 2015 | Caucasian | M/F | Obesity patients | TG, TC, LDL-C, HDL-C |
| Scherrer DZ [R77] | 2015 | Other ethnic | M/F | Healthy subjects | TC, LDL-C |
| Kolesnikova LI1 [R78] | 2015 | Caucasian | M/F | Healthy subjects | TG, TC, LDL-C, HDL-C |
| Kolesnikova LI2 [R78] | 2015 | Caucasian | M/F | Healthy subjects | TG, TC, LDL-C, HDL-C |
| Liang X1 [R79] | 2016 | Asian | M/F | Healthy subjects | TG, TC, LDL-C, HDL-C |
| Liang X2 [R79] | 2016 | Asian | M/F | Healthy subjects | TG, TC, LDL-C, HDL-C |
| Liang X3 [R79] | 2016 | Asian | M/F | Healthy subjects | TG, TC, LDL-C, HDL-C |
| Zhang CH [R80] | 2016 | Asian | M/F | CHD patients | TG, TC, Ox-LDL |
| Szpakowicz A [R81] | 2016 | Caucasian | M/F | CHD patients | TG, TC, LDL-C, HDL-C |
| Fridman O1 [R82] | 2016 | Caucasian | M/F | CHD patients | TG, TC, LDL-C, HDL-C |
| Fridman O2 [R82] | 2016 | Caucasian | M/F | Control subjects | TG, TC, LDL-C, HDL-C |
| Zargari M [R83] | 2016 | Caucasian | M/F | T2DM patients | HDL-C |
| Alharbi KK [R84] | 2016 | Caucasian | M/F | T2DM patients | TG, TC, LDL-C, HDL-C |
| Martínez-Quintana E [R85] | 2017 | Caucasian | M/F | CHD patients | TC, LDL-C |

*PON1*: Paraoxonase 1 gene; M: male; F: female; CHD: coronary heart disease; T2DM: type 2 diabetes mellitus; TG: triglycerides; TC: total cholesterol; LDL-C: low-density lipoprotein cholesterol; HDL-C: high-density lipoprotein cholesterol.

**Table S2:** Ox-LDL levels by the genotypes of the *PON1* rs662 polymorphism.

| **First author, reference** | **Genotype** | |  | **Ox-LDL, mg/L** | |
| --- | --- | --- | --- | --- | --- |
|  | **QQ** | **QR+RR** |  | **QQ** | **QR+RR** |
| Qian QW1 [R25] | 20 | 101 |  | 0.40±0.12 | 0.48±0.13 |
| Qian QW2 [R25] | 9 | 116 |  | 0.47±0.14 | 0.58±0.17 |
| Qian QW3 [R25] | 9 | 41 |  | 0.45±0.09 | 0.43±0.12 |
| Qian QW4 [R25] | 25 | 102 |  | 0.33±0.15 | 0.35±0.14 |
| Qian SH1 [R26] | 13 | 52 |  | 0.48±0.10 | 0.48±0.13 |
| Qian SH2 [R26] | 10 | 55 |  | 0.49±0.10 | 0.54±0.13 |
| Qian SH3 [R26] | 14 | 51 |  | 0.33±0.09 | 0.35±0.31 |
| Qian SH1 [R38] | 20 | 80 |  | 0.42±0.10 | 0.44±0.13 |
| Qian SH2 [R38] | 22 | 79 |  | 0.37±0.09 | 0.43±0.13 |
| Qian SH3 [R38] | 27 | 78 |  | 0.33±0.09 | 0.41±0.41 |
| Lakshmy R1 [R59] | 42 | 82 |  | 0.38±0.20 | 0.33±0.14 |
| Lakshmy R2 [R59] | 108 | 113 |  | 0.31±0.13 | 0.3±0.11 |
| Zhang CH [R80] | 38 | 62 |  | 1.9±0.03 | 2±0.07 |

*PON1*: Paraoxonase 1 gene; Ox-LDL: oxidized low density lipoprotein.

**Table S3: Plasma lipid levels by the genotypes of the *PON1* rs662 polymorphism**

| **First author, reference** | **Number** | |  | **TG, mmol/L** | |  | **TC, mmol/L** | |  | **LDL-C, mmol/L** | |  | **HDL-C, mmol/L** | |
| --- | --- | --- | --- | --- | --- | --- | --- | --- | --- | --- | --- | --- | --- | --- |
|  | **QQ** | **QR+RR** |  | **QQ** | **QR+RR** |  | **QQ** | **QR+RR** |  | **QQ** | **QR+RR** |  | **QQ** | **QR+RR** |
| Ruiz J [R1] | 207 | 227 |  | 1.82±1.2 | 1.89±1.2 |  | 5.87±1.2 | 5.93±1.1 |  | - | - |  | 1.19 ± 0.4 | 1.27±0.4 |
| Antikainen M [R2] | 211 | 169 |  | 1.60±0.63 | 1.7±0.67 |  | 5.16±0.64 | 5.21±0.63 |  | 3.62±0.53 | 3.62±0.54 |  | 0.82±0.14 | 0.81±0.14 |
| Suehiro T [R3] | 20 | 114 |  | 1.33±0.68 | 1.59±0.69 |  | 4.81±0.78 | 5.03±0.95 |  | 2.95±0.78 | 3.14±0.95 |  | 1.24±0.31 | 1.16±0.32 |
| Odawara M [R4] | 26 | 138 |  | 1.54 ± 0.84 | 1.59 ± 1.05 |  | 5.30 ± 1.15 | 5.12 ± 0.99 |  | - | - |  | 1.25 ± 0.35 | 1.25 ± 0.36 |
| Sanghera DK1 [R5] | 111 | 114 |  | 1.35±0.68 | 1.38±0.73 |  | 5.99±1.28 | 5.91±1.17 |  | 4.16±1.15 | 4.12±1.02 |  | 1.14±0.35 | 1.1±0.34 |
| Sanghera DK2 [R5] | 76 | 365 |  | 1.27±0.65 | 1.22±0.59 |  | 5.68±1.19 | 5.76±1.24 |  | 3.65±1.1 | 3.71±1.23 |  | 1.36±0.36 | 1.42±0.43 |
| Pati N [R6] | 30 | 90 |  | - | - |  | - | - |  | - | - |  | 1.31±0.22 | 1.22±0.31 |
| Ombres D [R7] | 227 | 246 |  | 1.84±1.21 | 2.07±1.41 |  | 5.45±1.21 | 5.58±1.1 |  | 3.51±1.05 | 3.60±0.94 |  | 1.13±0.3 | 1.07±0.31 |
| Ko YL [R8] | 30 | 188 |  | 1.58±0.86 | 1.71±2.11 |  | 5.29±0.9 | 5.32±1.13 |  | 3.21±0.74 | 3.27±0.88 |  | 1.37±0.42 | 1.32±0.41 |
| Hasselwander O [R9] | 77 | 88 |  | - | - |  | 6.11±1.17 | 6.11±1.53 |  | 3.77±1.03 | 3.79±1.37 |  | 1.10± 0.35 | 1.16± 0.49 |
| Singh S [R10] | 136 | 54 |  | 1.8±0.7 | 1.74±0.47 |  | 4.04±1.15 | 4.15±2.08 |  | 2.17±0.93 | 2.21±0.82 |  | 1.06±0.3 | 0.97±0.2 |
| Dessi M [R11] | 88 | 108 |  | 1.37± 0.58 | 1.47± 0.63 |  | 5.59±1.06 | 5.51±1.23 |  | 3.54 ± 0.98 | 3.51±1.15 |  | 1.4±0.34 | 1.31±0.31 |
| Pfohl M [R12] | 139 | 149 |  | - | - |  | 4.94 ± 1.00 | 4.94 ± 1.09 |  | 2.93 ± 0.83 | 2.94 ± 0.89 |  | 1.02 ± 0.36 | 1.01± 0.32 |
| MacKness B1 [R13] | 71 | 97 |  | - | - |  | 6.46±1.14 | 6.76±3.28 |  | 4.71±0.98 | 4.69±1.06 |  | 0.96±0.29 | 1.02±0.31 |
| MacKness B2 [R13] | 106 | 80 |  | - | - |  | 6±0.86 | 6.35±0.97 |  | 4.32±0.73 | 4.63±0.94 |  | 1·19±0·35 | 1.23±0.34 |
| Aubó C [R14] | 238 | 228 |  | - | - |  | 5.89±1.17 | 5.7±1.21 |  | 4.05±1.01 | 3.9±1.08 |  | 1.17±0.4 | 1.17±0.36 |
| Imai Y [R15] | 46 | 327 |  | 1.50±0.76 | 1.57±0.95 |  | 5.12±0.80 | 5.25±0.94 |  | 3.14±0.80 | 3.22±0.91 |  | 1.29±0.31 | 1.30±0.36 |
| Gardemann A [R16] | 1420 | 1364 |  | 1.72±1.02 | 1.7±0.94 |  | 5.43±1.09 | 5.43±1.11 |  | - | - |  | - | - |
| Sen-Banerjee S1 [R17] | 230 | 262 |  | 2.74±1.21 | 2.69±1.22 |  | 5.04±1.06 | 5.04±1.01 |  | - | - |  | 0.91± 0.28 | 0.96± 0.36 |
| Sen-Banerjee S2 [R17] | 279 | 239 |  | 2.32±1.13 | 2.35±1.41 |  | 5.2±1.01 | 5.15±1.03 |  | - | - |  | 1.09±0.31 | 1.09±0.28 |
| Turban S1 [R18] | 92 | 89 |  | 1.89±0.69 | 1.79±0.64 |  | 5.76±0.67 | 5.69±0.59 |  | 3.75±0.53 | 3.75±0.51 |  | 1.14±0.32 | 1.1±0.3 |
| Turban S2 [R18] | 84 | 88 |  | 1.77±0.63 | 1.77±0.59 |  | 5.66±0.54 | 5.72±0.74 |  | 3.75±0.47 | 3.73±0.57 |  | 1.11±0.25 | 1.19±0.31 |
| Liu R1 [R19] | 28 | 90 |  | 1.88± 1.07 | 1.68±0.84 |  | 6.58± 1.16 | 6.56±1.08 |  | 2.60±0.69 | 2.35±0.81 |  | 1.35± 0.36 | 1.26± 0.45 |
| Liu R2 [R19] | 25 | 103 |  | 1.22± 0.43 | 1.24±0.36 |  | 4.95± 0.88 | 5.06± 0.67 |  | 2.84± 0.81 | 3.09±0.64 |  | 1.38± 0.23 | 1.37± 0.45 |
| Tomás M [R20] | 308 | 346 |  | - | - |  | 5.86±1.09 | 5.71± 1.17 |  | 3.97±0.99 | 3.86±1.08 |  | 1.26 ± 0.35 | 1.22±0.36 |
| Koch M [R21] | 206 | 184 |  | - | - |  | 5.4±1.1 | 5.33±1.09 |  | 3.1± 0.9 | 3.1±0.9 |  | 1.2±0.4 | 1.21±0.41 |
| Yamada A [R22] | 28 | 209 |  | 1.22 ± 0.85 | 1.25±0.76 |  | 5.25 ±0.95 | 5.39±0.93 |  | - | - |  | 1.61 ±0.42 | 1.72±0.45 |
| Liu R1 [R23] | 23 | 105 |  | 3.92± 2.25 | 3.43± 1.55 |  | 5.33± 0.96 | 5.18± 0.94 |  | 2.51± 0.91 | 2.61± 0.77 |  | 0.98± 0.27 | 1.01± 0.25 |
| Liu R2 [R23] | 25 | 104 |  | 1.22± 0.43 | 1.23± 0.36 |  | 4.97± 0.88 | 5.24± 1.89 |  | 2.84± 0.80 | 3.1± 0.65 |  | 1.40± 0.23 | 1.38± 0.44 |
| Kuremoto K [R24] | 38 | 141 |  | 1.45±0.59 | 1.59±0.88 |  | 5.09±0.7 | 4.88±0.89 |  | - | - |  | 1.31±0.4 | 1.2±0.4 |
| Qian QW1 [R25] | 20 | 101 |  | 1.26±0.59 | 1.49±0.67 |  | 4.40 ±0.88 | 4.89±1.04 |  | 3.81±1.17 | 3.66±1.18 |  | 1.33±0.22 | 1.2±0.34 |
| Qian QW2 [R25] | 9 | 116 |  | 2.13±1.20 | 2.54±1.35 |  | 5.15±0.68 | 5.67±0.84 |  | 3.93±1.35 | 3.91±1.38 |  | 1.25±0.22 | 1.09±0.31 |
| Qian QW3 [R25] | 9 | 41 |  | 2.16 ±0.87 | 2.14 ±0.86 |  | 5.37 ±0.98 | 5.38 ±0.86 |  | 3.85 ±1.26 | 3.84 ±1.32 |  | 1.14 ±0.35 | 1.10 ±0.4 |
| Qian QW4 [R25] | 25 | 102 |  | 1.07 ±0.40 | 1.07 ±0.41 |  | 4.37 ±0.70 | 4.35 ±0.94 |  | 3.46 ±1.03 | 2.98 ±0.78 |  | 1.35 ±0.31 | 1.35 ±0.33 |
| Qian SH1 [R26] | 13 | 52 |  | 2.47±1.13 | 2.49±1.2 |  | 6.64±1.00 | 6.65±1.1 |  | 4.49±1.49 | 4.52±1.51 |  | 1.06±0.22 | 1.02±0.26 |
| Qian SH2 [R26] | 10 | 55 |  | 1.80±0.87 | 2.24±1.15 |  | 5.15±0.66 | 5.52±1.27 |  | 3.81±0.88 | 3.97±1.15 |  | 0.92±0.21 | 0.87±0.22 |
| Qian SH3 [R26] | 14 | 51 |  | 1.07±0.40 | 1.1±0.48 |  | 4.37±0.63 | 4.36±0.65 |  | 3.26±1.03 | 3.31±0.78 |  | 1.35±0.21 | 1.34±0.33 |
| Robertson KS [R27] | 879 | 864 |  | - | - |  | - | - |  | - | - |  | 0.80± 0.24 | 0.81± 0.25 |
| Ueno T [R28] | 46 | 142 |  | 1.86±1.1 | 1.53±1.09 |  | 5.37±1.01 | 5.23±0.96 |  | - | - |  | 1.19±0.28 | 1.32±0.54 |
| Hu Y [R29] | 30 | 122 |  | 1.16±0.57 | 1.58±0.92 |  | 4.10±0.70 | 5.02±1.17 |  | 2.00±0.41 | 2.4±0.7 |  | 1.20±0.30 | 1.16±0.25 |
| Campo S [R30] | 132 | 168 |  | - | - |  | - | - |  | - | - |  | 1.44 ±0.28 | 1.43 ±0.22 |
| Xiao ZJ [R31] | 95 | 549 |  | 1.45± 0.64 | 1.47± 0.81 |  | 4.82± 0.96 | 4.78± 0.99 |  | 2.80± 0.72 | 2.80± 0.81 |  | 1.44± 0.40 | 1.41± 0.39 |
| Agachan B1 [R32] | 100 | 107 |  | 1.79±0.85 | 1.71±1.16 |  | 5.09±1.29 | 4.88±1.3 |  | 3.15±0.96 | 2.81±1.11 |  | 1.07±0.34 | 1.1±0.43 |
| Agachan B2 [R32] | 36 | 71 |  | 1.54±0.99 | 1.46±0.61 |  | 4.1±0.94 | 4.44±0.79 |  | 2.87±1.05 | 3.07±0.68 |  | 1.03±0.37 | 1.1±0.43 |
| Srinivasan SR1 [R33] | 612 | 620 |  | 1.46±1.03 | 1.6±1.56 |  | - | - |  | 3.17 ± 0.9 | 3.15± 0.89 |  | 1.21±0.32 | 1.23±0.33 |
| Srinivasan SR2 [R33] | 65 | 489 |  | 0.98±0.51 | 1.08±0.79 |  | - | - |  | 3.11 ± 1 | 2.97±0.93 |  | 1.51±0.52 | 1.35±0.39 |
| Li SY [R34] | 44 | 93 |  | 1.44 ±0.68 | 1.54 ±0.99 |  | 5.10 ±0.74 | 5.23 ±0.93 |  | - | - |  | - | - |
| Oliveira SA [R35] | 327 | 398 |  | 2.04±1.59 | 1.9±1.57 |  | 5.55±1.17 | 5.38±1.08 |  | 3.53±0.99 | 3.41±0.99 |  | 1.08±0.36 | 1.12±0.37 |
| Xiao ZJ [R36] | 137 | 882 |  | 1.58±0.89 | 1.58±0.93 |  | 4.82±1.01 | 4.81±1.02 |  | 2.84±0.75 | 2.84±0.83 |  | 1.39±0.38 | 1.35±0.38 |
| Blatter Garin MC1 [R37] | 373 | 337 |  | - | - |  | - | - |  | 3.84±1.16 | 3.95±1.06 |  | 1.14 ±0.19 | 1.1 ±0.19 |
| Blatter Garin MC2 [R37] | 88 | 111 |  | - | - |  | - | - |  | 3.6±1.03 | 3.84±0.95 |  | 1.28 ±0.28 | 1.30 ±0.29 |
| Qian SH1 [R38] | 20 | 80 |  | 2.37±0.85 | 2.44±1.12 |  | 5.65±0.65 | 5.71±0.46 |  | 4.81±0.78 | 4.79±0.91 |  | 1.28±0.18 | 1.23±0.23 |
| Qian SH2 [R38] | 22 | 79 |  | 2.27±1.06 | 2.4±1.22 |  | 5.34±1.01 | 5.42±1.11 |  | 3.28±1.24 | 3.96±1.46 |  | 0.98±0.20 | 1.02±0.25 |
| Qian SH3 [R38] | 27 | 78 |  | 1.03±0.42 | 1.06±0.48 |  | 4.34±0.63 | 4.36±0.63 |  | 3.06±0.91 | 3.09±1.01 |  | 0.36±0.91 | 1.34±0.34 |
| Aydin M1 [R39] | 14 | 51 |  | 1.84±0.6 | 1.91±0.61 |  | 5.16±1.31 | 5.21±1.39 |  | 3.38±1.15 | 3.29±1.05 |  | 1.15±0.43 | 1.09±0.3 |
| Aydin M2 [R39] | 38 | 46 |  | 1.37± 0.59 | 1.56± 0.45 |  | 4.07±0.85 | 4.13±0.87 |  | 2.85±0.49 | 3.18±0.4 |  | 1.08±0.29 | 1.07±0.26 |
| Manresa JM [40] | 657 | 707 |  | - | - |  | 5.82± 1.14 | 5.72± 1.14 |  | 3.9 ±1.03 | 3.85 ±1.03 |  | 1.37±0.38 | 1.35± 0.4 |
| Huang Q [R41] | 15 | 138 |  | 1.65 ± 0.67 | 1.59 ± 0.98 |  | 4.10 ± 0.92 | 4.3 ±0.84 |  | 2.77 ± 0.45 | 2.57±0.82 |  | 1.35 ±0.64 | 1.25±1.59 |
| Juretić D1 [R42] | 42 | 52 |  | 2.27 ± 1.43 | 3.09 ± 1.23 |  | 6.00 ± 1.27 | 5.98 ± 1.66 |  | 3.81 ± 1.10 | 3.60 ± 0.96 |  | 1.25 ± 0.28 | 1.22 ± 0.25 |
| Juretić D2 [R42] | 40 | 41 |  | 2.29 ± 1.74 | 2.34 ± 1.41 |  | 5.84 ± 1.07 | 6.51 ± 1.39 |  | 3.38 ± 0.90 | 4.17 ± 1.22 |  | 1.44 ± 0.30 | 1.39 ± 0.30 |
| Juretić D3 [R42] | 18 | 10 |  | 1.32 ± 0.49 | 1.08 ± 0.28 |  | 5.34 ± 0.74 | 5.38 ± 0.65 |  | 3.35 ± 0.70 | 3.56 ± 0.67 |  | 1.38 ± 0.33 | 1.34 ± 0.30 |
| Juretić D4 [R42] | 55 | 31 |  | 1.17 ± 0.80 | 1.04 ± 0.34 |  | 5.57 ± 0.77 | 5.51 ± 0.65 |  | 3.53 ± 0.66 | 3.56 ± 0.58 |  | 1.50 ± 0.28 | 1.47 ± 0.41 |
| Rios DL1 [R43] | 124 | 137 |  | 2.00±1.09 | 1.69±0.92 |  | - | - |  | - | - |  | 0.67±0.19 | 0.72±0.2 |
| Rios DL2 [R43] | 75 | 101 |  | 1.84±1.68 | 2.04±1.15 |  | - | - |  | - | - |  | 0.81±0.20 | 0.83±0.22 |
| Rios DL3 [R43] | 63 | 92 |  | 1.93±1.55 | 1.98±1.12 |  | - | - |  | - | - |  | 0.71±0.17 | 0.73±0.21 |
| Rios DL4 [R43] | 34 | 86 |  | 1.53±0.91 | 1.57±0.66 |  | - | - |  | - | - |  | 0.83±0.25 | 0.85±0.21 |
| Lahiry P [R44] | 332 | 973 |  | - | - |  | 5.55±0.38 | 5.59±0.39 |  | 3.62±0.35 | 3.65±0.37 |  | - | - |
| Irace C1 [R45] | 64 | 54 |  | 1.77±0.76 | 1.68±0.88 |  | 5.17±1.06 | 5.38±1.01 |  | - | - |  | 1.22±0.34 | 1.24±0.21 |
| Irace C2 [R45] | 26 | 39 |  | 1.04±0.44 | 1.73±1.56 |  | 5.97±0.72 | 5.56±1.03 |  | - | - |  | 1.89±0.59 | 1.53±0.52 |
| Irace C3 [R45] | 27 | 24 |  | 1.74±0.5 | 1.49±0.7 |  | 5.61±0.85 | 5.22±0.78 |  | - | - |  | 1.29±0.39 | 1.24±0.23 |
| Irace C4 [R45] | 37 | 30 |  | 1.8±0.86 | 1.91±1.03 |  | 4.86±1.09 | 5.48±1.24 |  | - | - |  | 1.19±0.34 | 1.22±0.18 |
| Fu R [R46] | 56 | 180 |  | 1.86 ± 0.68 | 1.88 ± 0.72 |  | 6.89 ± 1.20 | 6.87 ± 1.13 |  | 3.30 ± 0.65 | 3.28 ± 0.69 |  | 1.36 ± 0.48 | 1.35 ± 0.4 |
| Garcés C [R47] | 624 | 642 |  | 0.81± 0.29 | 0.83 ±0.3 |  | 4.73 ±0.74 | 4.72 ±0.69 |  | 2.81 ±0.69 | 2.81 ±0.66 |  | 1.54±0.33 | 1.54 ±0.35 |
| Guxens M [R48] | 1125 | 1204 |  | - | - |  | 5.72 ± 1.06 | 5.78 ± 1.11 |  | 3.05 ± 0.74 | 3.1 ± 0.8 |  | 1.19 ± 0.34 | 1.24 ± 0.36 |
| van Himbergen TM [R49] | 785 | 729 |  | - | - |  | - | - |  | - | - |  | 1.6±0.4 | 1.6±0.4 |
| van den Berg SW [R50] | 284 | 282 |  | - | - |  | 5.2 ± 0.93 | 5.23 ± 1.03 |  | 3.3 ± 0.83 | 3.33 ± 0.88 |  | 1.16 ± 0.33 | 1.23 ± 0.36 |
| Regieli JJ [R51] | 420 | 374 |  | - | - |  | 6.07± 0.87 | 6.0 ±0.88 |  | 4.36 ±0.78 | 4.26± 0.79 |  | 0.92± 0.23 | 0.93± 0.22 |
| Porntadavity S [R52] | 23 | 137 |  | 1.41 + 0.44 | 1.39+ 0.48 |  | 5.21 + 0.55 | 5.26 + 0.49 |  | 3.11 + 0.43 | 3.09+ 0.41 |  | 1.46 + 0.27 | 1.54+ 0.33 |
| Birjmohun RS [R53] | 1640 | 1531 |  | - | - |  | - | - |  | 4.1±1.0 | 4.12±1.0 |  | 1.33±0.40 | 1.34±0.39 |
| Aydin M [54] | 109 | 240 |  | 1.33 ± 0.58 | 1.67 ± 0.9 |  | 4.5 ± 1.18 | 4.74± 1.2 |  | 2.73± 0.94 | 2.97± 0.93 |  | 1.19 ± 0.31 | 1.05 ± 0.28 |
| Gluba A [R55] | 221 | 193 |  | - | - |  | 5.62±1.55 | 5.47±1.22 |  | 2.79±1.79 | 2.92±1.69 |  | 1.32±0.49 | 1.22±0.34 |
| Mohamed RH [R56] | 41 | 159 |  | 1.66±0.26 | 1.85±0.28 |  | 5.31±0.85 | 5.83±0.89 |  | 3.24±0.84 | 3.82±0.88 |  | 1.36±0.16 | 1.16±0.17 |
| Likidlilid A1 [R57] | 16 | 87 |  | 1.98±0.91 | 1.95±1.1 |  | 7.47±1.18 | 7.14±1.07 |  | 5.39±1.42 | 4.97±1.14 |  | 1.18±0.32 | 1.28±0.34 |
| Likidlilid A2 [R57] | 24 | 79 |  | 1.23±0.59 | 1.25±0.51 |  | 4.67±0.46 | 4.59±0.51 |  | 2.88±0.55 | 2.77±0.63 |  | 1.33±0.43 | 1.28±0.35 |
| Chen XJ [R58] | 23 | 187 |  | - | - |  | 5.03±0.85 | 5.25± 1.75 |  | 2.25±0.28 | 3.06 ±0.56 |  | 1.14 ±0.37 | 1.16±0.42 |
| Lakshmy R1 [R59] | 42 | 82 |  | - | - |  | - | - |  | - | - |  | 1.07 ± 0.26 | 1± 0.26 |
| Lakshmy R2 [R59] | 108 | 113 |  | - | - |  | - | - |  | - | - |  | 1.03± 0.26 | 1.01 ± 0.25 |
| Altuner D1 [R60] | 65 | 35 |  | 1.79 ± 1.04 | 1.92± 0.9 |  | 5.33± 1.21 | 5.15±1.16 |  | 3.2 ± 0.91 | 3.53± 0.95 |  | 1.26 ± 0.26 | 1.24±0.21 |
| Altuner D2 [R60] | 38 | 12 |  | 1.31 ± 0.79 | 1.52± 0.77 |  | 4.82 + 0.85 | 5.61+ 1.38 |  | 2.96 ± 0.86 | 3.44 ± 1.34 |  | 1.19 ± 0.2 | 1.2 ± 0.26 |
| Trenk D [R61] | 384 | 376 |  | - | - |  | 5.15±1.16 | 5.12±1.19 |  | - | - |  | - | - |
| Vaisi-Raygani A1 [R62] | 157 | 123 |  | 1.89 ± 0.95 | 2.01 ± 1.07 |  | 4.77 ± 1.24 | 4.82 ± 0.82 |  | 2.42 ± 0.78 | 2.43 ± 0.63 |  | 1.24 ± 0.29 | 1.22 ± 0.2 |
| Vaisi-Raygani A2 [R62] | 92 | 42 |  | 1.65 ± 0.68 | 1.38 ± 0.53 |  | 4.74 ± 0.67 | 4.7 ± 0.75 |  | 2.25 ± 0.47 | 2.26 ± 0.47 |  | 1.26 ± 0.17 | 1.31 ± 0.13 |
| Sibbing D [R63] | 812 | 712 |  | - | - |  | - | - |  | 2.59±0.96 | 2.59±1.01 |  | 1.32±0.39 | 1.34±0.39 |
| Wang Y1 [R64] | 43 | 363 |  | 1.11±0.47 | 1.38±1.13 |  | 4.33±0.72 | 4.41±0.86 |  | 2.44±0.66 | 2.56±0.78 |  | 1.48±0.43 | 1.38±0.35 |
| Wang Y2 [R64] | 54 | 287 |  | 0.93±0.39 | 1.06±1.09 |  | 4.10±0.70 | 4.26±0.72 |  | 2.25±0.55 | 2.37±0.64 |  | 1.50±0.31 | 1.52±0.32 |
| Haj Mouhamed D1 [R65] | 99 | 63 |  | - | - |  | - | - |  | - | - |  | 0.90 ± 0.25 | 0.96 ± 0.24 |
| Haj Mouhamed D2 [R65] | 103 | 35 |  | - | - |  | - | - |  | - | - |  | 1.00 ± 0.16 | 1.03 ± 0.32 |
| Moura LM [R66] | 45 | 273 |  | - | - |  | 6.21±1.27 | 5.63±1.37 |  | 4.18±1.1 | 3.68±1.11 |  | 1.17±0.21 | 1.33±0.3 |
| Yang J [R67] | 17 | 153 |  | - | - |  | - | - |  | 2.8±1.0 | 2.59±0.7 |  | 0.96±0.24 | 1.01±0.33 |
| Murugan MM1 [R68] | 122 | 198 |  | 2±0.62 | 1.97±0.74 |  | 5.31± 0.12 | 5.23±1.05 |  | 3.43±1.2 | 3.37±0.99 |  | 0.97±0.18 | 0.96 ±0.18 |
| Murugan MM2 [R68] | 179 | 151 |  | 1.82± 0.37 | 1.72±0.42 |  | 4.46± 0.49 | 4.46 ±0.49 |  | 2.61± 0.51 | 2.62 ±0.49 |  | 1.02±0.12 | 1.04±0.13 |
| Kucuk ST1 [R69] | 57 | 78 |  | 1.8±0.73 | 1.7±0.89 |  | 5.21±1.27 | 5.12±1.15 |  | 3.4±1.06 | 3.36±0.93 |  | 0.97±0.23 | 0.96±0.25 |
| Kucuk ST2 [R69] | 43 | 67 |  | 1.39±0.96 | 1.39±1.17 |  | 5.69±1.33 | 5.43±1.26 |  | 3.41±1.19 | 3.22±1.08 |  | 1.41±0.39 | 1.17±0.42 |
| Hassan MA [R70] | 100 | 129 |  | 1.5 ± 0.85 | 1.63± 0.86 |  | 4.4±0.95 | 4.73 ± 1.0 |  | 3.4 ± 0.99 | 3.53 ± 1.0 |  | 1.2 ± 0.35 | 0.93± 0.33 |
| Fekih O1 [R71] | 23 | 93 |  | 0.75±0.26 | 0.71 ± 0.31 |  | 3.83±0.46 | 3.97 ± 0.69 |  | 1.99 ± 0.49 | 2.34 ± 0.60 |  | 1 .47 ± 0.36 | 1.42 ± 0.32 |
| Fekih O2 [R71] | 20 | 71 |  | 0.79±0.61 | 0.83± 0.51 |  | 3.10±0.73 | 3.36 ± 0.65 |  | 1.68 ± 0.55 | 1.84 ± 0.48 |  | 1.24 ± 0.44 | 1.22 ± 0.44 |
| Shao ZY [R72] | 85 | 500 |  | 1. 84±0. 88 | 1. 77±0.89 |  | 4. 63±1. 38 | 4. 33±1. 11 |  | 3. 45±0. 85 | 3. 34±0. 93 |  | 1. 26±0. 43 | 1. 19±0. 40 |
| Bortolasci CC [R73] | 151 | 184 |  | - | - |  | - | - |  | - | - |  | 1.24±0.37 | 1.22±0.38 |
| Macharia M [R74] | 133 | 711 |  | 1.5 ±1.0 | 1.5 ±0.94 |  | 5.6 ±1.2 | 5.56 ±1.2 |  | 3.7 ±1.0 | 3.66±1.0 |  | 1.3 ±0.4 | 1.3±0.35 |
| Bounafaa A [R75] | 113 | 92 |  | 1.74±0.74 | 2.01±0.77 |  | 4.61±1.28 | 4.76±0.96 |  | - | - |  | - | - |
| Alegría-Torres JA [R76] | 45 | 72 |  | 0.94± 0.36 | 0.91 ±0.26 |  | 4.23±0.89 | 4.33±0.63 |  | 2.77±0.67 | 2.64±0.65 |  | 1.32±0.33 | 1.25±0.25 |
| Scherrer DZ [R77] | 109 | 475 |  | - | - |  | 4.55± 0.78 | 4.45± 0.79 |  | 2.61 ± 0.57 | 2.6 ± 0.63 |  | - | - |
| Kolesnikova LI1 [R78] | 23 | 42 |  | 0.89 ± 0.34 | 0.73± 0.38 |  | 4.32 ± 0.58 | 4.27± 0.73 |  | 2.75 ± 0.54 | 2.72 ± 0.57 |  | 1.21 ± 0.19 | 1.22 ± 0.19 |
| Kolesnikova LI2 [R78] | 11 | 43 |  | 0.54 ± 0.23 | 0.4 ± 0.15 |  | 3.44 ± 0.78 | 3.48 ± 0.92 |  | 2.02 ± 0.63 | 2.17 ± 0.74 |  | 1.17 ± 0.17 | 1.18 ± 0.15 |
| Liang X1 [R79] | 22 | 129 |  | 1.28±0.60 | 1.2± 0.88 |  | 4.71±0.29 | 4.89±0.13 |  | 2.62±0.16 | 2.76±0.11 |  | 1.47±0.16 | 1.55±0.27 |
| Liang X2 [R79] | 17 | 181 |  | 1.12± 0.64 | 1.23±0.99 |  | 4.83±0.23 | 4.93±0.11 |  | 2.67±0.18 | 2.66±0.08 |  | 1.60±0.12 | 1.71±0.08 |
| Liang X3 [R79] | 11 | 165 |  | 1.66± 1.47 | 1.77± 0.78 |  | 4.66±0.71 | 4.86±0.2 |  | 2.47±0.41 | 2.55±0.16 |  | 0.88±0.23 | 1.21±0.13 |
| Zhang CH [R80] | 38 | 62 |  | 1.65 ±0.08 | 1.9 ±0.11 |  | 6.71±0.19 | 6.82 ±0.22 |  | - | - |  | - | - |
| Szpakowicz A [R81] | 332 | 302 |  | 1.4 ±0.75 | 1.44± 0.83 |  | 5.05± 1.13 | 5.07±1.06 |  | 3.31 ±0.99 | 3.32 ±0.95 |  | 1.13± 0.36 | 1.14 ±0.32 |
| Fridman O1 [R82] | 77 | 49 |  | 2.24±2.11 | 1.93±1.54 |  | 5.31±1.93 | 5.0±1.61 |  | 2.73±1.23 | 2.44±0.98 |  | 1.53±0.35 | 1.48±0.35 |
| Fridman O2 [R82] | 116 | 87 |  | 1.58±1.18 | 1.71±1.49 |  | 4.96±1.4 | 5.17±1.03 |  | 2.71±1.08 | 2.67±0.84 |  | 1.59±0.43 | 1.73±0.47 |
| Zargari M [R83] | 72 | 43 |  | - | - |  | - | - |  | - | - |  | 1.49±0.34 | 1.43±0.33 |
| Alharbi KK [R84] | 149 | 251 |  | 2.1±1.1 | 2.51±1.29 |  | 5.0±0.9 | 5.05±0.98 |  | 3.6±0.8 | 3.68±0.82 |  | 0.6±0.2 | 0.6±0.2 |
| Martínez-Quintana E [R85] | 245 | 284 |  | - | - |  | 4.24±1.17 | 4.19±1.1 |  | 2.41±0.9 | 2.4±0.89 |  | - | - |

*PON1*: Paraoxonase 1 gene; TG: triglycerides; TC: total cholesterol; LDL-C: low-density lipoprotein cholesterol; HDL-C: high-density lipoprotein cholesterol.
